# A Biologically Constrained, Mathematical Model of Cortical Wave Propagation Preceding Seizure Termination

**DOI:** 10.1371/journal.pcbi.1004065

**Published:** 2015-02-17

**Authors:** Laura R. González-Ramírez, Omar J. Ahmed, Sydney S. Cash, C. Eugene Wayne, Mark A. Kramer

**Affiliations:** 1 Department of Mathematics and Statistics, Boston University, Boston, Massachusetts, United States of America; 2 Department of Neurology, Massachusetts General Hospital, Boston, Massachusetts, United States of America; 3 Harvard Medical School, Boston, Massachusetts, United States of America; University of Toronto, CANADA

## Abstract

Epilepsy—the condition of recurrent, unprovoked seizures—manifests in brain voltage activity with characteristic spatiotemporal patterns. These patterns include stereotyped semi-rhythmic activity produced by aggregate neuronal populations, and organized spatiotemporal phenomena, including waves. To assess these spatiotemporal patterns, we develop a mathematical model consistent with the observed neuronal population activity and determine analytically the parameter configurations that support traveling wave solutions. We then utilize high-density local field potential data recorded *in vivo* from human cortex preceding seizure termination from three patients to constrain the model parameters, and propose basic mechanisms that contribute to the observed traveling waves. We conclude that a relatively simple and abstract mathematical model consisting of localized interactions between excitatory cells with slow adaptation captures the quantitative features of wave propagation observed in the human local field potential preceding seizure termination.

## Author Summary

Nearly 50 million people worldwide suffer from epilepsy, a chronic neurological condition characterized by recurrent, unprovoked seizures. Although some clinical and biological principles of seizures are known, many aspects of spontaneous human seizures remain poorly understood. Recordings from electrodes placed directly on and within the brain provide a unique view of seizure activity, and have revealed specific brain voltage patterns associated with this pathological state. In particular, there is evidence that organized waves of activity propagate over the brain during a seizure. However, quantitatively characterizing and understanding the mechanisms that support these waves remains an open challenge. The goal of this work is to address this challenge through a combination of mathematical modeling and clinical recordings. Through this interdisciplinary approach, we seek to understand general features that support the spatiotemporal patterns of seizure termination. We propose that a relatively simple and abstract mathematical model consisting of localized interactions of closely neighboring excitatory cells with slow adaptation can support the propagation of the waves found in clinical recordings. Improved understanding of the mechanisms supporting seizure activity promises novel developments in treatment strategies tailored to the observed activity of individual patients.

## Introduction

Epilepsy is a dynamical disease [1] that manifests in many ways, including as organized patterns of brain voltage activity during a seizure. In general, a patient’s epilepsy may be classified through established clinical and imaging procedures and, based on the classification, a treatment strategy may be developed [2]. Although pharmacological and surgical treatment of epilepsy often succeeds, the exact mechanisms that lead to different kinds of epilepsy and produce a seizure are still largely unknown; common proposed biological mechanisms include altered interactions between excitatory and inhibitory neurons [3, 4] and hyperexcitation [5]. Although the underlying mechanisms that initiate and support the seizure may widely vary [6], some manifestations of the seizure remain stereotyped, including clinical symptoms and voltage dynamics [2]. For human patients, one of the most common observations of brain activity during seizure consists of chronic voltage recordings. These invasive or noninvasive observations provide detailed spatiotemporal information about the *in vivo* voltage dynamics of spontaneous seizures. Invasive local field potential (LFP) recordings provide fine spatial resolution of brain voltage activity during seizure, and have recently led to new insights [7–9].

LFP recordings are thought to represent the active ionic and synaptic currents within a volume of cortical tissue; in this way, the LFP captures the aggregate activity of large neuronal populations [10–12]. In healthy and diseased brain tissue, wave-like spatiotemporal activity has been observed in the field activity of many systems including the olfactory system of invertebrates [13] and vertebrates [14, 15], turtle visual cortex [16–20], rat visual cortex [21–23], rat hippocampus [24], rat somatosensory cortex [25], monkey motor cortex [26], human motor cortex [27], and human retina [28].

Coordinated spatiotemporal activity is thought to serve a functional role in computation and communication between subsystems of the brain. For example, waves are thought to support synaptic modification during development, as observed in the visual system (e.g., [28, 29]). Although seizure activity is characterized by stereotyped voltage rhythms [30, 31] and coupling between rhythms across space [7, 32], the role of spatiotemporal patterns (e.g., waves [21]) remains an active research area [33, 34]. Moreover, the biological mechanisms that support these manifestations of seizure remain incompletely understood; further understanding these features promises improved therapies for epilepsy, in addition to a deeper understanding of organized neuronal population activity in brain function and dysfunction.

In addition to clinical and experimental recordings, computational models provide an alternative, powerful approach to investigate the biological mechanisms that support observed brain voltage activity. In general, the combination of experimental data and mathematical modeling has proved useful in understanding propagation dynamics in the brain. For example, experimental observations made in a cultured one-dimensional slice agree with a theoretical framework based on an integrate-and-fire model [35, 36], and the compression and reflection of visually evoked cortical waves [37] has been modeled in [38]. Both animal models (e.g., [39]) and computational models (e.g., [6, 40]) permit controlled, detailed observations of a given seizure process, and the ability to accurately manipulate this process. Importantly, unlike typical observations from clinical recordings, models permit a detailed accounting of the biological mechanisms that support the observed activity. However, the starting assumptions of a model oversimplify the biological processes of the *in vivo* brain (e.g., removal of a brain region from the surrounding network, or omission of some cell types). An exact relationship between these models and human epilepsy is often difficult to determine. Clinical observations and models therefore provide different insights into seizure activity. Clinical recordings provide accurate *in vivo* observations of spontaneous seizures from human patients, yet the biological mechanisms that support this activity remain predominantly unknown. Models provide detailed control and manipulations of candidate biological mechanisms, but the relationship to spontaneous seizures in humans remains unknown. Ideally, a unified procedure would exploit the advantages of each approach and mitigate the disadvantages. Implementing this type of procedure linking human clinical recordings to mechanisms in an abstract and simple mathematical model is one goal of this paper.

We propose to characterize invasive clinical voltage recordings from small regions of human cortex preceding seizure termination through comparison with a mathematical model. To do so, we simulate the cooperative synaptic transmembrane current found in clinical LFP recordings using a relatively simple and abstract mathematical mean field model. Mean field neural models, or neural fields, are used to represent coarse-grained variables in space, consisting of thousands of interconnected neurons (i.e., spanning approximately a few hundred micrometers) [41, 42]. Models of neural fields have a long history in computational neuroscience [21, 43–45], and have been successfully employed in many areas, including the study of spatiotemporal dynamics [46–51], with features such as periodic patterns [52], bumps and multi-bumps [53, 54], and waves [37, 44, 55–57]. Because these models are expressed as differential-integral equations, mathematical theory exists to rigorously analyze the model behavior. Here we undertake a mathematical analysis of a mean field model consistent with the observed LFP data to obtain the exact solution for traveling wave dynamics, and deduce parameter relationships that support wave propagation. We then constrain the model solutions using features of LFP recordings of traveling wave dynamics preceding seizure termination observed in a population of human subjects during seizure. In particular, by using the observed width and speed of the LFP waves we obtain parameter estimates consistent with known biological features of cortex, namely timescales and the synaptic connectivity profile. We show that a relatively simple mathematical model consisting of a population of excitatory neurons with localized interactions and an adaptation term is sufficient to mimic the observed LFP waves preceding seizure termination. In this way, the proposed framework links clinical recordings with mathematical models to propose candidate mechanisms supporting a poorly understood aspect of seizure activity: the spatiotemporal dynamics preceding seizure termination in a small patch of human cortex.

## Results

Our goal is to isolate and characterize in a relatively abstract mathematical model the mechanisms that support the emergence of traveling wave dynamics preceding seizure termination. To do so, we first characterize these dynamics as observed in invasive brain voltage recordings from a population of human subjects during seizure. We show that stereotypical traveling wave patterns emerge in the LFP with consistent quantitative features. Then, we implement an activity-based mathematical model of neural population dynamics. We obtain explicit traveling wave solutions for the model together with conditions that ensure the existence of a wave of given speed and width. We then further constrain the model parameters using the wave features observed in the *in vivo* LFP data. Finally, we use these model results to propose candidate mechanisms that support the observed traveling wave activity preceding seizure termination.

### Analysis of *in vivo* LFP data during seizure reveals traveling wave dynamics

#### Description of clinical data

LFP data were collected from three patients: (Patient 1) a 32 year old male with cortical dysplasia and mesial temporal sclerosis, (Patient 2) a 45 year old male with unknown etiology, and (Patient 3) a 21 year old male with a dysplastic lesion. The data were recorded using the NeuroPort array (Blackrock Microsystems, Salt Lake City, UT) which, as in previous studies [7, 8], consisted of a 4 mm by 4 mm microelectrode array composed of 100 platinum-tipped silicon probes (either 1.0 or 1.5 mm long shanks). In each subject, the array was placed in an area of cortex which was expected to be resected at the time of definitive surgery, 1–3 cm outside of the nominal seizure focus as determined from electrocorticography, but well within an area to which the seizure rapidly spread. Recordings were made from 96 active electrodes and data were sampled at 30 kHz (0.3–7 kHz bandwidth). LFP data were extracted by bandpass filtering the original recordings from 2–50 Hz (fourth-order Butterworth, zero-phase digital filtering) and downsampling to 5000 Hz. All patients were enrolled after informed consent was obtained and approval was granted for these studies by local Institutional Review Boards.

#### Illustration of traveling waves during seizure

**Figure 1:**
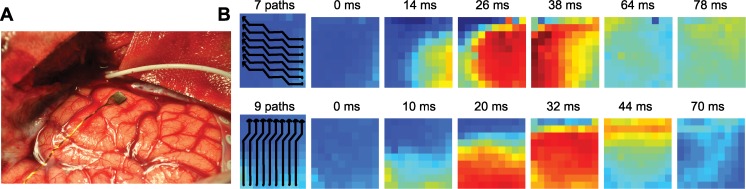
Illustrations of one-dimensional wave propagation. **(a)** Example of the 4 mm by 4 mm microelectrode array (black, in center of figure) implanted in human cortex. **(b)** Each subfigure displays the spatial pattern of the LFP activity recorded from a 10-by-10 microelectrode array at different times. Warm (cool) colors indicate high (low) voltage values (standardized) for two instances of wave propagation in the top and bottom rows. For each wave different one-dimensional paths (black lines in leftmost columns) beginning at the filled circles capture the wave propagation across the microelectrode array. **(Top row)** Visual inspection suggests a wave of organized activity during Seizure 2 (Patient 1) that propagates from the lower right corner of the microelectrode array to the upper left corner. **(Bottom row)** A wave of organized activity during Seizure 1 (Patient 1) that propagates from the lower part of the microelectrode array to the upper part.

We analyzed the LFP data recorded during three seizures from Patient 1; we labeled these “Seizure 1”, “Seizure 2” and “Seizure 3”. From Patient 2 we analyzed two seizures, labeled “Seizure 4” and “Seizure 5”; and from Patient 3 we analyzed three seizures labeled “Seizure 6”, “Seizure 7” and “Seizure 8”. In all cases, we focused on LFP data recorded near seizure termination; the data for Seizure 1, Seizure 2 and Seizure 3 began approximately 31 s, 47 s and 43 s, respectively, before seizure termination and lasted 18 s. Within this time interval, we observed numerous traveling waves, which consisted of transient, large amplitude organized patterns of LFP activity that propagated across the microelectrode array (two example wave events are shown in Fig. 1). Within Seizures 1, 2, and 3, we observed 40, 41, and 59 waves events, respectively. The data for Seizure 4 and Seizure 5 began approximately 44 s and 43 s respectively before seizure termination and lasted 20 s and 18 s, respectively. Within Seizures 4 and 5, we observed 33 and 52 traveling waves, respectively. The data for Seizure 6, Seizure 7, and Seizure 8 began approximately 21 s, 19 s, and 27 s, respectively, before seizure termination and lasted 12 s, 18 s, and 26 s, respectively. Within Seizures 6, 7, and 8, we observed 49, 53, and 61 traveling waves, respectively. Traveling waves of increased LFP activity were observed in all recordings (see Methods). Visual inspection of these waves (Fig. 1) suggested that propagation was dominated by movement in one spatial direction, and that this dominant movement appeared at each wave event. An algorithm was constructed to estimate the one-dimensional path of propagation for each wave across the microelectrode array (see Methods). The one-dimensional motion of each wave, as illustrated in Fig. 1, formed the basis for subsequent analysis. Other types of propagation were also found in the clinical recordings but not included in the following analysis (for details, see Methods).

#### Estimation of wave features

**Figure 2:**
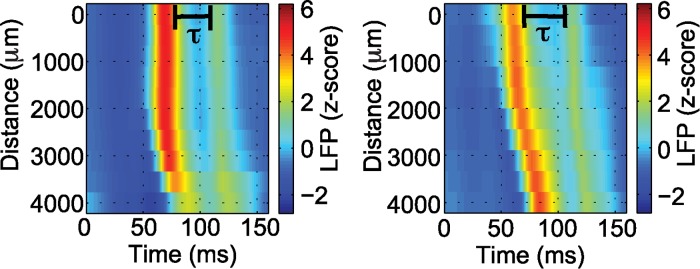
Illustration of two large amplitude waves followed by a reverberation of activity. The waves are plotted in one-dimensional space (vertical axis) as a function of time. The one-dimensional path extends across the two-dimensional microelectrode array (examples in Fig. 1). This large amplitude wave is followed by a subsequent “reverberation”- a smaller amplitude wave (yellow or green in color). The horizontal black line indicates the reverberation time (τ). Warm (cool) colors indicate high (low) voltage values; scale bar at right.

**Figure 3:**
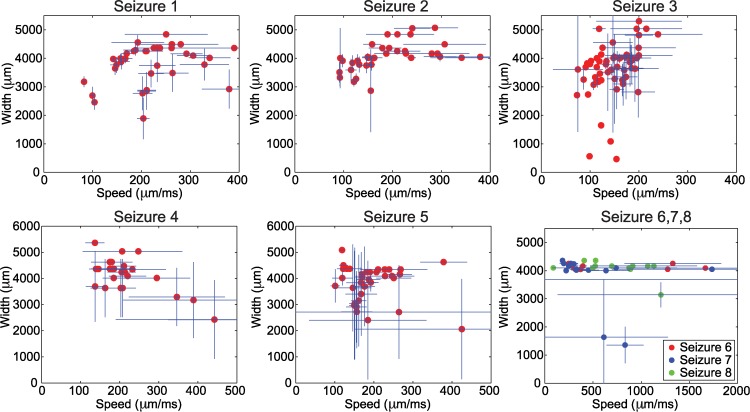
Width versus speed of the traveling wave activity for the three patients. Each subplot shows the mean width and mean speed of each wave (red dots) together with a 90% confidence interval for both width and speed for each wave (vertical and horizontal blue lines). The confidence intervals are computed for each wave over the replicates of one-dimensional paths established for each wave (Fig. 1). In some cases, the existence of different one-dimensional paths produces a broad confidence interval for the estimate of the speed and width. In other cases, the existence of a unique one-dimensional path, or the estimation of the same quantity from the different one-dimensional paths, produces a narrow confidence interval.

**Figure 4:**
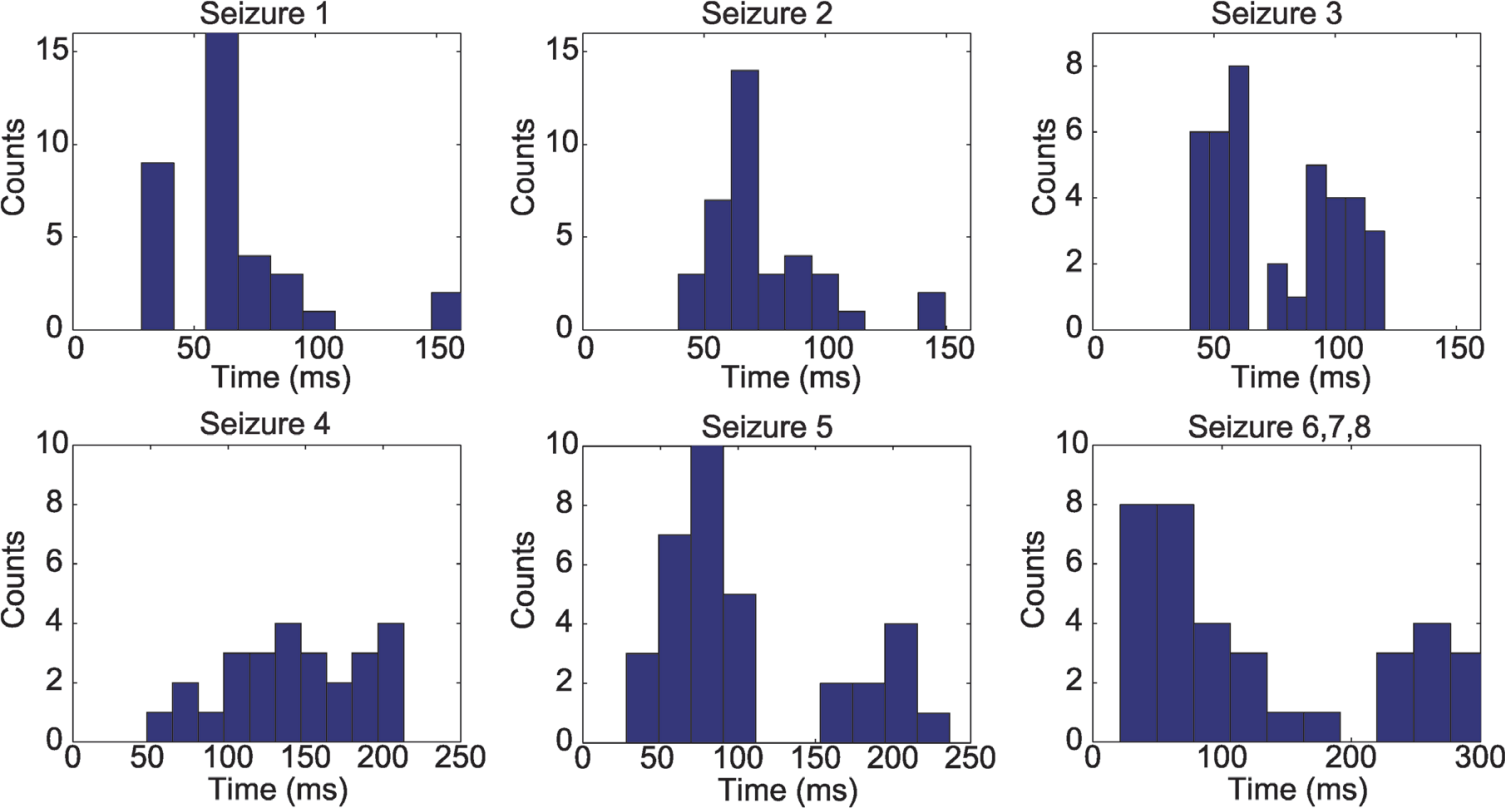
Reverberation time histograms of the seizure activity for the three patients. Each subplot shows the number of occurrences (or “counts”) of each reverberation time. For Seizures 1, 2 and 3 the maximum number of counts is between 60 to 70 ms; for Seizure 4, the values are broadly distributed between 100 and 200 ms; for Seizure 5 between 70 to 100 ms; and for Seizure 5, 6 and 7 between 40 to 70 ms.

We focused our analysis on two fundamental features of the one-dimensional wave propagation: speed and width. The speed was computed by determining the evolution in time of a point with large voltage amplitude across ten different electrodes along the one-dimensional path of the wave through the micro electrode array. Width was computed by estimating the spatial extent (i.e., the number of electrodes) exceeding a fixed, large amplitude (see Methods). In addition to these two fundamental wave features, we also determined the time interval between the initial, large amplitude wave propagation across the microelectrode array and a subsequent, smaller amplitude fluctuation or “reverberation” (examples in Fig. 2), we label this the “reverberation time” (see Methods). We show the results for the wave speeds and widths of all the analyzed waves in Fig. 3, and the reverberation times histograms in Fig. 4. These results are summarized for each seizure in Table 1 where we show the mean speeds, mean widths, and mean reverberation times for the waves analyzed in each seizure. For each wave, we computed the mean values of the quantity of interest (speed, width, or reverberation time) over the different one-dimensional paths. The minimum/maximum values correspond to the minimum/maximum obtained for the mean values of the quantity over the total of number of waves analyzed for a given seizure. We note that for Patient 3 the data analysis was restricted due to the quality of the recordings (see Methods).

**Table 1:** Mean speeds, mean widths, and mean reverberation times for the waves analyzed in each seizure.

Seizure number	Speed (*μ*m/ms)	Width (*μ*m)	Reverberation time (ms)
1	[82, 389]	[1891, 4841]	[29, 161]
2	[91, 380]	[2361, 5069]	[39, 150]
3	[86, 240]	[2815, 5621]	[40, 120]
4	[137, 506]	[2411, 5361]	[48, 213]
5	[101, 424]	[2059, 5085]	[28, 237]
6, 7 and 8	[89, 2416]	[1360, 4360]	[22, 304]

In this table, [a,b] indicate the minimum (a) and the maximum (b) of the corresponding measurement over the total number of waves analyzed for a given seizure.

For the three seizures of Patient 1 we observed consistent ranges of speed (≈ 80–380 *μ*m/ms), width (≈ 1900–5600 *μ*m), and reverberation times (≈ 30–150 ms). This suggests that similar, one-dimensional wave propagation patterns occur in the three seizures from this patient. For the two seizures of Patient 2, we also observed consistent ranges of speed (≈ 100–500 *μ*m/ms), width (≈ 2000–5300 *μ*m), and reverberation times (≈ 30–230 ms). Finally, for the three seizures of Patient 3, we observed broader ranges of speed (≈ 90–2400 *μ*m/ms), width (≈ 1300–4300 *μ*ms), and reverberation times (≈ 20–300 ms). These characterizations of the *in vivo* wave dynamics provide information about the clinical observations, however an important question remains: what biological mechanisms support this traveling wave activity preceding seizure termination in the LFP? We propose to begin addressing this question in the next section through the inclusion of a mathematical model.

### A mathematical model of traveling wave dynamics in LFP recordings

The mechanisms that produce organized neuronal population activity are extremely complex [58]. In an effort to characterize and understand the neuronal population activity observed in the clinical recordings preceding seizure termination, we implement here a relatively simple neural field model [59]. The biophysical basis for these types of models are understood by considering the interaction of a finite number of synaptically coupled neurons. Many different formulations for neural fields exist [60], with implications for the interpretation of the model variables and parameters. These different mathematical formulations of neural field models can be broadly separated into two categories: voltage-based formulations, and activity-based formulations [59]. In a voltage-based model, the time scale of the dynamics is related to the membrane properties of the post-synaptic cells, while in an activity-based-model, the time scale of the dynamics is related to the synaptic decay [59]. We choose the latter formulation here. In its simplest form, the activity-based model is one of the most basic models to arise in mathematical neuroscience [61]. Beyond this simple form, activity-based models have been extended to include additional features (e.g., absolute refractoriness [41, 62]). In addition, the activity-based model is consistent with the notion that the LFP dynamics are dominated by the time scale of synaptic effects [10, 63], and activity-based models have been proposed as more realistic than voltage-based models [64, 65]. We note that most mathematical analysis of neural field models utilizes the voltage-based formulation [44, 65, 66]. In particular, in [67] the authors performed a complete analysis of the existence and stability of traveling wave solutions in the voltage-based formulation. To the best of our knowledge, a mathematical analysis of traveling wave existence and stability in an activity-based model with adaption has not been performed.

We now develop a one-dimensional model to describe important features of the neuronal population activity observed *in vivo*. The choice of a one-dimensional model is motivated by the observation that a majority of traveling waves observed in the LFP recordings travel in approximately one-dimension, with features as described in the previous section. To simplify the model, we consider only a single population of excitatory neurons. In doing so, we will show that—in the mathematical model—inhibitory neurons are not required to mimic features of the observed LFP data immediately preceding seizure termination. To prevent the activity from remaining in a permanent excited state, which will give rise to a front solution (see Methods), we include an adaptation term that directly regulates the activity. This adaptation accounts for a natural process that will drive the population activity back to a rest state. From the mathematical point of view, adding this adaptation term permits traveling pulse solutions in the model consistent with key features of the clinical recordings. As we describe, using this relatively abstract and simple activity-based model with an adaptation term, we are able to replicate the reverberation observed in the LFP recordings.

The specific neural field model we employ is

$$\begin{array}{l} u_{t}\left(x,t\right)=-\alpha u\left(x,t\right)+\alpha H\left(\frac{1}{2\sigma }\int_{-\infty }^{+\infty }e^{-\frac{|x-y|}{\sigma }}u\left(y,t\right)dy+P(x,t)-k\right)-\alpha \beta_{0}q\left(x,t\right)\\ q_{t}\left(x,t\right)=\delta u\left(x,t\right)-\delta q\left(x,t\right), \end{array}$$ (1)

where *u*(*x*, *t*) is the mean synaptic activity, *q*(*x*, *t*) is the adaptation, and *P*(*x*, *t*) is an external input, all evaluated at position *x* and time *t*. In particular, we consider that *u*(*x*, *t*) represents the activity of a cortical column with extent less than 20 *μ*m situated at position *x* and time *t*. We interpret *u*(*x*, *t*), a dimensionless quantity, as the deviation from a baseline of activity. Therefore, *u*(*x*, *t*) = 0 represents a resting state of activity, and negative values represent a depression of resting activity [41]. We note that “negative activity” (i.e., a reduction in activity below the baseline rate) in one region reduces the input received in neighboring regions. In this formulation, we interpret the adaption term, *q*(*x*, *t*), as representing a local homeostatic regulation mechanism that evolves on a slower timescale than *u*(*x*, *t*) and acts to maintain the activity near a target baseline. When the activity *u*(*x*, *t*) falls below the baseline value (i.e., *u*(*x*, *t*) < 0), the adaption *q*(*x*, *t*) decreases which acts to increase *u*(*x*, *t*). Conversely, when the activity increases above baseline (i.e., *u*(*x*, *t*) > 0), the adaption *q*(*x*, *t*) increases and acts to decrease *u*(*x*, *t*). We note that homeostatic regulation mechanisms act on a variety of timescales, including relatively short timescales (on the order of seconds) [68]. *H* is the Heaviside function, which becomes non-zero when the synaptic input exceeds a synaptic threshold *k*:

$$H\left(x-k\right)=\{\begin{array}{ll} 1 & \text{if}x\geq k\\ 0 & \text{if}x<k. \end{array}$$

We note that the adaptation term in (1) is located outside of the Heaviside function. In this phenomenological model with a simple adaptive scheme, the adaptation term acts as a local feedback mechanism to depress the synaptic drive. This model is motivated by the linear negative feedback proposed in [44]. We note that, in voltage-based models, different formulations for adaption exist; these include negative feedback both inside the threshold function [44, 51, 69] and outside of the threshold function [49, 53]. We show in S1 Text of Supporting Information that the model (1) updated to include the adaption term inside of the Heaviside function does not produce damped oscillations; instead, the traveling wave solution returns monotonically to rest after excitation. This monotonic evolution is inconsistent with the reverberation observed in the LFP data of interest here (examples in Fig. 2).

There are 5 parameters in the model (1). Each possesses a biological interpretation: *α* is the decay rate parameter for the synaptic activity term, *δ* is the decay rate parameter for the adaptation term, *σ* is the spatial rate of decay of connectivity, *k* is the synaptic input threshold, and *β*_0_ accounts for the strength of the adaptation term on the synaptic dynamics. For simplicity we set *β* = *α β*_0_. Both time and space units were scaled to represent milliseconds and microns, respectively (see Methods). There are two additional parameters that we employ in the subsequent analysis: *c* is the wave speed, and *w* is the wave width. These parameters are not directly specified in the model, but instead are features of the traveling wave dynamics.

Our goal is to identify the parameter configurations that support traveling waves in this model consistent with the observed LFP activity. In particular, we are interested in solutions that support only one extremum of high amplitude activity, so called pulses, as these have been characterized using the LFP data. To that end, we first determine under what parameter configurations traveling waves of high amplitude activity exist in the model. To do so, we rewrite the equations in a moving coordinate frame *z* = *x*-*ct*; this frame is moving with a constant speed *c*. By identifying the stationary solutions of this system, we determine solutions that move with a constant speed *c*, and a constant width *w*, without changing their shape: so called traveling waves. Depending on the model parameters, we find that the linearization of the associated system in the moving coordinate frame consists of either purely real or complex eigenvalues. The explicit traveling wave solutions for both the real and imaginary case are now considered. We state the solutions here; detailed analysis may be found in Methods.

#### Traveling Wave Solution: Real Eigenvalues Case

**Figure 5:**
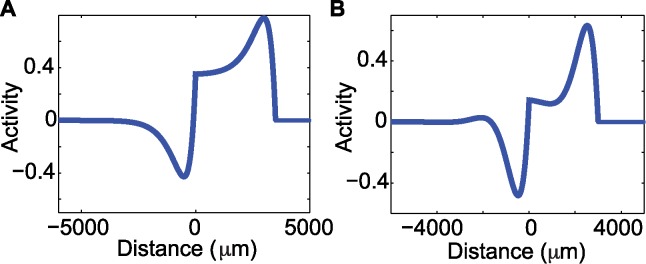
Analytic solution for the traveling pulse in the real and complex eigenvalue case. **(a)** The onset of the pulse consists of a rapid increase in activity, followed by a rapid decrease due to adaptation, and then a monotonic return to rest (zero activity). In this gure α = 20/s, δ = 2/s, β = 1.5, σ = 200 μm, *c* = 180 μm/ms and *ω* = 3500 μm. **(b)** In the complex eigenvalue case there is a pulse followed by a depression of activity due to the adaptation term. Unlike the solution in the real eigenvalue case, damped oscillations follow this depression as activity returns to the rest state. In this figure α = 20/s, δ = 2/s, β = 4.6, σ = 160 μm, *c* = 250 μm/ms and *w* = 3000 μm.

We begin by considering the case in which the eigenvalues of the associated linear system of (1) are purely real. This occurs when $\beta <\frac{{\left(\alpha -\delta \right)}^{2}}{4\delta }$ (see Methods), and the traveling wave solution of the activity of width *w* and speed *c* in the moving coordinate frame *z* = *x*—*ct* is:

$$u(z)=\{\begin{array}{ll} 0 & \text{if}z\geq w\\ \frac{\alpha }{\delta (\alpha +\beta )(\lambda_{+}-\lambda_{-})}\left(\lambda_{-}e^{\lambda_{+}(z-w)}(\delta -c\lambda_{+})-\lambda_{+}e^{\lambda_{-}(z-w)}(\delta -c\lambda_{-})+\delta (\lambda_{+}-\lambda_{-})\right) & \\ & \text{if}0<z<w\\ \frac{\alpha }{\delta (\alpha +\beta )(\lambda_{+}-\lambda_{-})}\left(\lambda_{-}(e^{-w\lambda_{+}}-1)(\delta -c\lambda_{+})e^{\lambda_{+}z}+\lambda_{+}(1-e^{-w\lambda_{-}})(\delta -c\lambda_{-})e^{\lambda_{-}z}\right) & \\ & \text{if}z\leq 0, \end{array}$$

where $\lambda_{\pm }=\frac{1}{2c}(\alpha +\delta \pm \sqrt{{\left(\alpha +\delta \right)}^{2}-4\delta (\alpha +\beta )})$. In this traveling wave solution (Fig. 5a), a pulse is followed by a depression of activity; this depression is due to the adaptation term. The activity then returns to a rest state after this depression in a monotonic fashion.

#### Traveling Wave Solution: Complex Eigenvalues Case

We now consider the case in which $\beta >\frac{{\left(\alpha -\delta \right)}^{2}}{4\delta }$. In this scenario, the linearization of the associated system of (1) contains imaginary eigenvalues, and the traveling wave solution of the activity of width *w* and speed *c* in the moving coordinate frame *z* = *x*-*ct* is:

$$u(z)=\{\begin{array}{ll} 0 & \text{if}z\geq w\\ \frac{\alpha }{\alpha +\beta }+\frac{2\alpha \sqrt{\beta }e^{\left(\frac{\alpha +\delta }{2c})(z-w\right)}}{\sqrt{\left(\alpha +\beta )(4\beta \delta -{\left(\delta -\alpha \right)}^{2}\right)}}\text{sin}(\frac{\sqrt{4\delta \beta -{\left(\alpha -\delta \right)}^{2}}}{2c}z+\phi_{1}) & \text{if}0<z<w\\ \frac{2\alpha \sqrt{\beta }e^{\left(\frac{\alpha +\delta }{2c})(z\right)}}{\sqrt{\left(\alpha +\beta )(4\beta \delta -{\left(\delta -\alpha \right)}^{2}\right)}}\;D\;\text{cos}(\frac{\sqrt{4\delta \beta -{\left(\alpha -\delta \right)}^{2}}}{2c}z+\phi_{2}) & \text{if}z\leq 0, \end{array}$$

where

$D=\sqrt{1-2e^{-w(\frac{\alpha +\delta }{2c})}\;\text{cos}(\frac{\sqrt{4\delta \beta -{\left(\alpha -\delta \right)}^{2}}}{2c}w)+e^{-w(\frac{\alpha +\delta }{c})}}$
$\phi_{1}={\text{tan}}^{-1}(\frac{A_{1}}{A_{2}})+\{\begin{array}{ll} \pi & \text{if}A_{1}<0\\ 0 & \text{if}A_{1}>0 \end{array},\phi_{2}={\text{tan}}^{-1}(\frac{A_{3}}{A_{4}})+\{\begin{array}{ll} \pi & \text{if}A_{4}<0\\ 0 & \text{if}A_{4}>0 \end{array}$
$A_{1}=(2\beta +\alpha -\delta )\text{sin}(\frac{\sqrt{4\delta \beta -{\left(\alpha -\delta \right)}^{2}}}{2c}w)-\sqrt{4\delta \beta -{\left(\delta -\alpha \right)}^{2}}\text{cos}(\frac{\sqrt{4\delta \beta -{\left(\alpha -\delta \right)}^{2}}}{2c}w)$
$A_{2}=-(2\beta +\alpha -\delta )\text{cos}(\frac{\sqrt{4\delta \beta -{\left(\alpha -\delta \right)}^{2}}}{2c}w)-\sqrt{4\delta \beta -{\left(\delta -\alpha \right)}^{2}}\text{sin}(\frac{\sqrt{4\delta \beta -{\left(\alpha -\delta \right)}^{2}}}{2c}w)$
$A_{3}=\sqrt{4\delta \beta -{\left(\alpha -\delta \right)}^{2}}+e^{-\frac{\alpha +\delta }{2c}w}A_{1}$
$A_{4}=(2\beta +\alpha -\delta )+e^{-\frac{\alpha +\delta }{2c}w}A_{2}.$

The solution for the complex eigenvalue case results in a pulse followed by a depression of activity due to the adaptation term. Unlike the solution in the real eigenvalue case, damped oscillations follow this depression as activity returns to the rest state (example in Fig. 5b). We note that the damped oscillations are dominated by a single positive deviation above rest, following the depression. This positive deviation is similar to the reverberation of activity following the traveling wave observed in the LFP (Fig. 2). We note (see S1 Text of the Supporting Information) that a different model with the adaption term included inside of the Heaviside function in (1) is unable to reproduce the damped oscillations observed in the LFP data.

#### Solution curves from matching conditions

**Figure 6:**
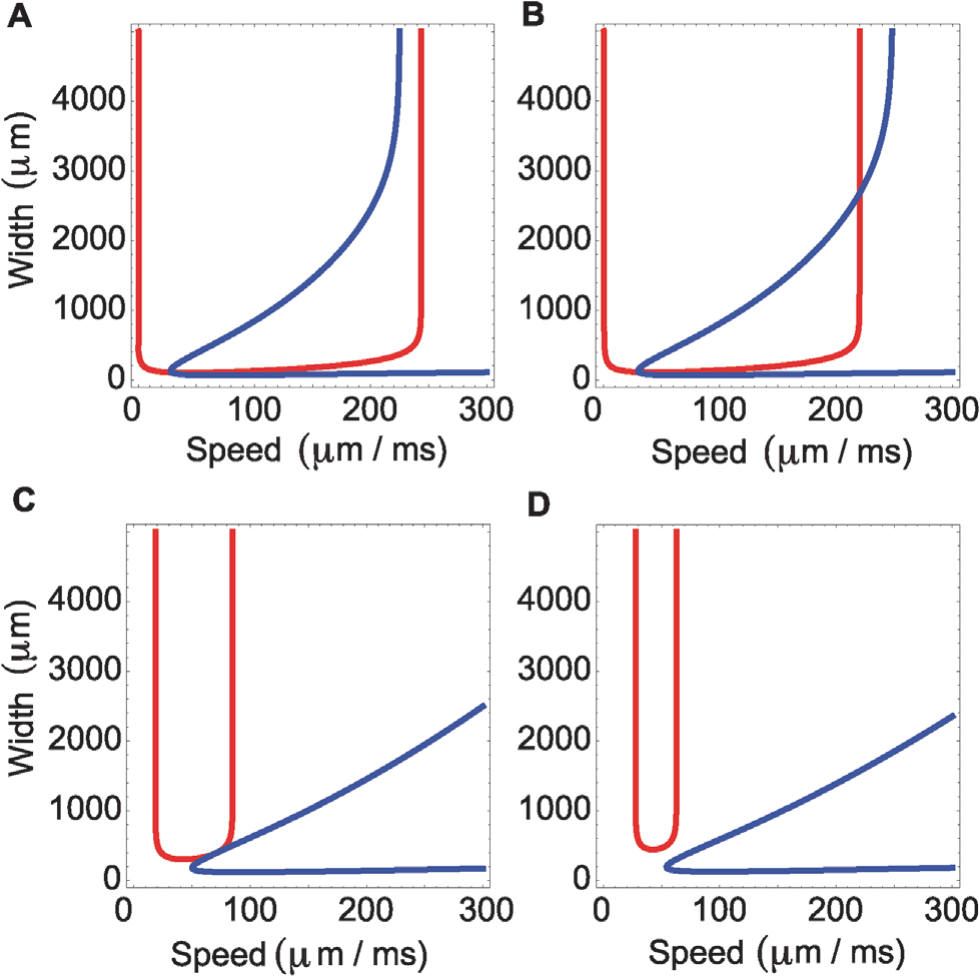
Width versus speed in the mathematical model for different values of the threshold *k*. The four subplots show the existence of waves given by the points of intersection of the matching conditions. The blue and red curves indicate the matching conditions at the points 0 and *ω*, respectively. We fix α = 25/s, α = 2.5/s, σ = 120 μm, β = 2.05, and by varying *k* we obtain the existence of no waves (d), one wave (a,c), or two waves (b). **(a)** The two curves intersect at a single point to specify a wave with speed 30 μm/ms and width 112 μm. **(b)** The two curves intersect at two points, resulting in a wave with speed 33 μm/ms and width 164 μm, and a wave with speed 220 μm/ms and width 2679 μm/ms. **(c)** The two curves intersect at a single point to specify a wave with speed 71 μm/ms and width 350 μm. **(d)** The two curves do not intersect, and therefore no wave solutions exist.

The interactions of neighboring cells affect the activity at a point *x*. In the presence of a pulse of high activity, such interactions reach the synaptic threshold *k* at exactly two points, say *x*_0_ and *x*_1_. The distance between *x*_0_ and *x*_1_ is the width of the wave *w* and the points *x* contained within (*x*_0_, *x*_1_) satisfy $\frac{1}{2\sigma }\int_{-\infty }^{+\infty }e^{-\frac{|x-y|}{\sigma }}u\left(y,t\right)dy>k$. At both *x*_0_ and *x*_1_, this inequality becomes an equality, i.e., the interaction term equals the synaptic threshold *k*. Equating the interaction terms at *x*_0_ and *x*_1_ defines the matching conditions. To simplify the analysis, and without loss of generality, we consider *x*_0_ = 0 and *x*_1_ = *w*. By fixing the parameters *α*, *δ*, *σ* and *β* and by setting the matching conditions to equal the same threshold *k* we obtain two curves, one for the position *x* = 0 (blue curves in Fig. 6) and the other for *x* = *w* (red curves in Fig. 6). In the *c*-*w* plane, the intersection of these curves determines the existence of traveling wave solutions to the model (1). Depending on the choice of parameters, there may exist no traveling waves, one traveling wave, or two traveling waves (examples in Fig. 6). We find that, for a solution with two traveling waves, one of the waves is slow and narrow, and the other wave is fast and wide (Fig. 6b).

**Figure 7:**
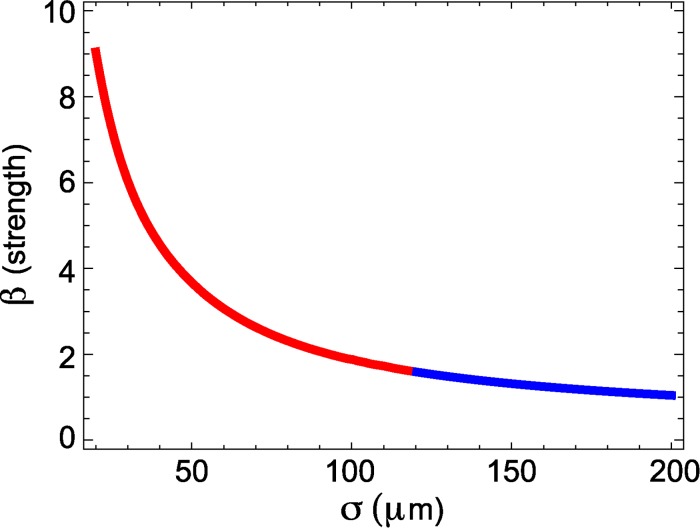
Solution curve in the σ-*β* plane obtained from the matching conditions in the mathematical model. We show the complex eigenvalues case (red) and the real eigenvalues case (blue). The parameters are *c* = 179 μm/ms, *w* = 3500 μm, α = 20/s, δ = 2/s. The curve shows pairs of *β* and *σ* for which a wave of speed *c* and width *ω* exist. By using the matching conditions we can determine the parameter *k* corresponding to a choice of *β* and *σ*.

If we consider instead fixed values of *c*, *w*, *α*, *δ* and solve the matching conditions, we obtain a solution curve in the *β*-*σ* plane that determines, if they exist, parameters *β* and *σ* for which we have a pulse with given speed *c* and width *w*. Moreover, by considering $k=\frac{1}{2\sigma }\int_{-\infty }^{+\infty }e^{-\frac{|y|}{\sigma }}u\left(y,t\right)dy$ or $k=\frac{1}{2\sigma }\int_{-\infty }^{+\infty }e^{-\frac{|w-y|}{\sigma }}u\left(y,t\right)dy$, we can solve for the threshold *k* corresponding to the choice of σ and β (for more details, see Methods). To illustrate the application of the matching conditions, we consider one typical traveling wave observed in the LFP recording with speed *c* = 179 *μ*m/ms and width *w* = 3500 *μ*m. For this example, we fix *α* = 20/s and *δ* = 2/s, and find a solution curve for the wave of the specified speed and width as a function of the two parameters *β* and *σ*. The solution curve consists of both real and imaginary parts (blue and red, respectively, in Fig. 7), corresponding to the real and complex eigenvalue cases of traveling wave solutions of the model (1). We note that all of the points along the solution curve satisfy the constraints of speed and width; additional constraints are required to select a single point on this curve.

### The period of the reverberation xes the model parameter

The mathematical model (1) contains five free parameters: *α*, *δ*, *σ*, *β* and *k*. In the previous section, we began restricting these parameters by establishing relationships between parameters that support traveling wave solutions. In particular, by fixing the time scales *α* and *δ*, together with a choice of speed *c* and width *w* deduced directly from the LFP data and hence constrained by the clinical observations, we may solve for the remaining parameters *β*, *σ*, and *k*. The matching conditions establish a relationship between *σ* and *β* (example in Fig. 7), and by choosing *β* and *σ* we can solve for the corresponding *k*, as described in the previous section. We now proceed to use the “reverberation” observed in the clinical data (examples in Fig. 2) to estimate the parameter *β* for each wave. In doing so, we will have used the clinical data and biophysical intuition to constrain further the model parameters.

**Figure 8:**
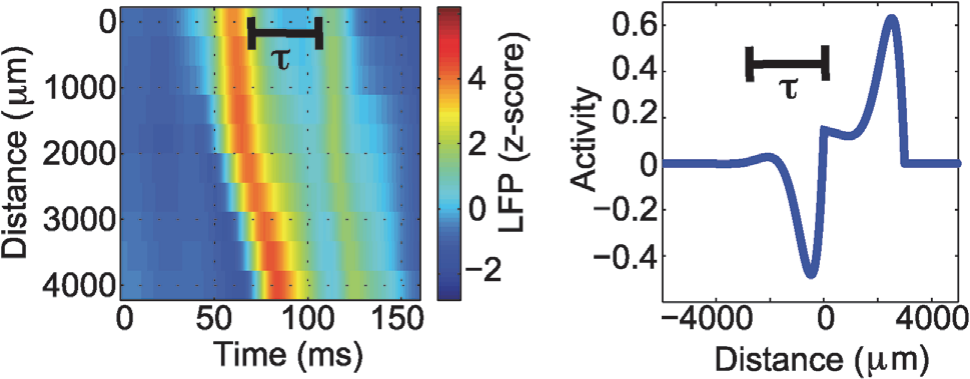
Illustration of the parameter β in the mathematical model estimated from the LFP data. Given a wave with a reverberation time of τ = 45 ms (left figure) and the approximate period of the complex eigenvalue pulse solution (right figure) we obtain a corresponding $\beta_{empirical}=\frac{1}{\delta }(\frac{4\pi^{2}}{\tau^{2}}+\frac{{\left(\delta -\alpha \right)}^{2}}{4})$.

Visual analysis of the *in vivo* LFP data shows that high amplitude pulses are followed by a reverberation, i.e., a secondary, smaller amplitude increase in activity (for more details, see Methods). Due to the nature of the traveling wave solutions, this feature is only present in the complex eigenvalue solution, i.e., when damped oscillations follow the pulse of high amplitude activity (example in Fig. 5b); we propose that the damped oscillations following the main pulse of the traveling wave mimic the reverberations observed in the LFP recordings. Hence, we restrict the following analysis to the complex eigenvalue case. We use the reverberation times estimated from the LFP data to fix the parameter *β* for each wave; we label these estimates *β*_*empirical*_. To do so, we set the periodic portion of the complex eigenvalue solution to possess a period consistent with the observed reverberation: given a reverberation time *τ* (example in Fig. 8), then $\beta_{empirical}=\frac{{\left(\delta -\alpha \right)}^{2}}{4\delta }+\frac{4\pi^{2}}{\delta \tau^{2}}$ (see Methods). In this way we constrain the model to replicate the period of the secondary bump (i.e., reverberation) present in the data (Fig. 8). Having done so, the model parameters *β*, *σ*, and *k* are now directly determined for each observed LFP wave.

#### Restriction of the ratio between activity and adaptation timescales

In the previous sections, we used features of the traveling wave data (the speed, width, and reverberation time) to constrain three model parameters: *β*, *σ*, and *k*. Two model parameters - *α* and *δ* - remain unconstrained. We now consider how different choices of the model timescales *α* and *δ* impact the existence of traveling wave solutions consistent with the LFP data. To do so we focus on two different orders of magnitude between the timescales and consider *α*/*δ* = 10 and *α*/*δ* = 100. These equations and the model (1) are consistent with the notion that adaptation (with timescale determined by *δ*) occurs more slowly than synaptic activity (with timescale determined by *α*). Moreover, once we fix the ratio *δ* = *α*/10 (or *δ* = *α*/100) we can estimate *c*, *w* and *β* from the clinical recordings and obtain *σ* and *k* from the matching conditions. Therefore, only a single free parameter remains: *α*. The rest of the parameters are constrained by either the clinical data or the matching conditions of the mathematical model.

**Figure 9:**
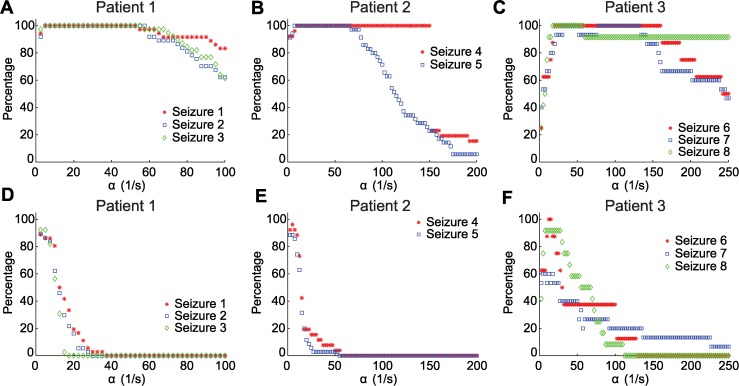
The percentage of waves from each seizure for which it is possible to find a physically reasonable solution (σ > 20μm) as the parameter α is varied. In the first row we fix δ = α/10, a difference of one order of magnitude between the timescales. In the second row we fix δ = *α*/100. **(a)** We note that for the three seizures of Patient 1 a value of *α* between 15/s and 53/s produces physically reasonable solutions for all analyzed waves. **(b)** For the two seizures of Patient 2 a value of between 15/s and 75/s produces physically reasonable solutions for 90% of the analyzed waves. **(c)** For all seizures of Patient 3, given *α* between 25/s and 150/s produces physically reasonable solutions for 90% of the analyzed waves. **(d-f)** At the ratio δ = *α*/100, the model solutions tend to be unphysical (i.e., σ becomes too small) for α >12/s. This analysis suggest that the model best replicates the observed LFP data when *δ* = *α*/10.

To characterize the impact of different choices of *α*, and the ratio *α*/*δ*, we fix both parameters in the model and determine whether the model supports wave activity consistent with the observed data and physical assumptions in the model. We therefore exclude solutions in which the matching conditions specify a connectivity extent (*σ*) of less than 20 *μ*m; these solutions are too small and inconsistent with the notion that the model (1) represents the activity *u* of coupled cortical columns. In Fig. 9 we show the percentage of waves for each seizure with a physically reasonable value of *σ* > 20 *μ*m for different choices of *α* and ratios *α/δ*. For all of the seizures from all three patients, we find that the model successfully reproduces the observed waves, and remains physically reasonable (*σ* *>* 20 *μ*m), for intermediate values of *α* and *δ* = *α*/10 (Fig. 9a–c). For a smaller value of *δ* = *α*/100, the model performs more poorly; i.e., the model produces more physically unreasonable solutions (Fig. 9d–f). We note that, for Patient 3, the waves are more difficult to reproduce compared to the other two patients. We conclude that the model best replicates the observed traveling waves in the LFP data preceding seizure termination when *δ* = *α*/10. At this ratio, a broad range of values in *α* exist that support physically reasonable solutions.

#### Relationship between adaptation timescale and model parameters *β*_0_, *k* and *σ*

**Figure 10:**
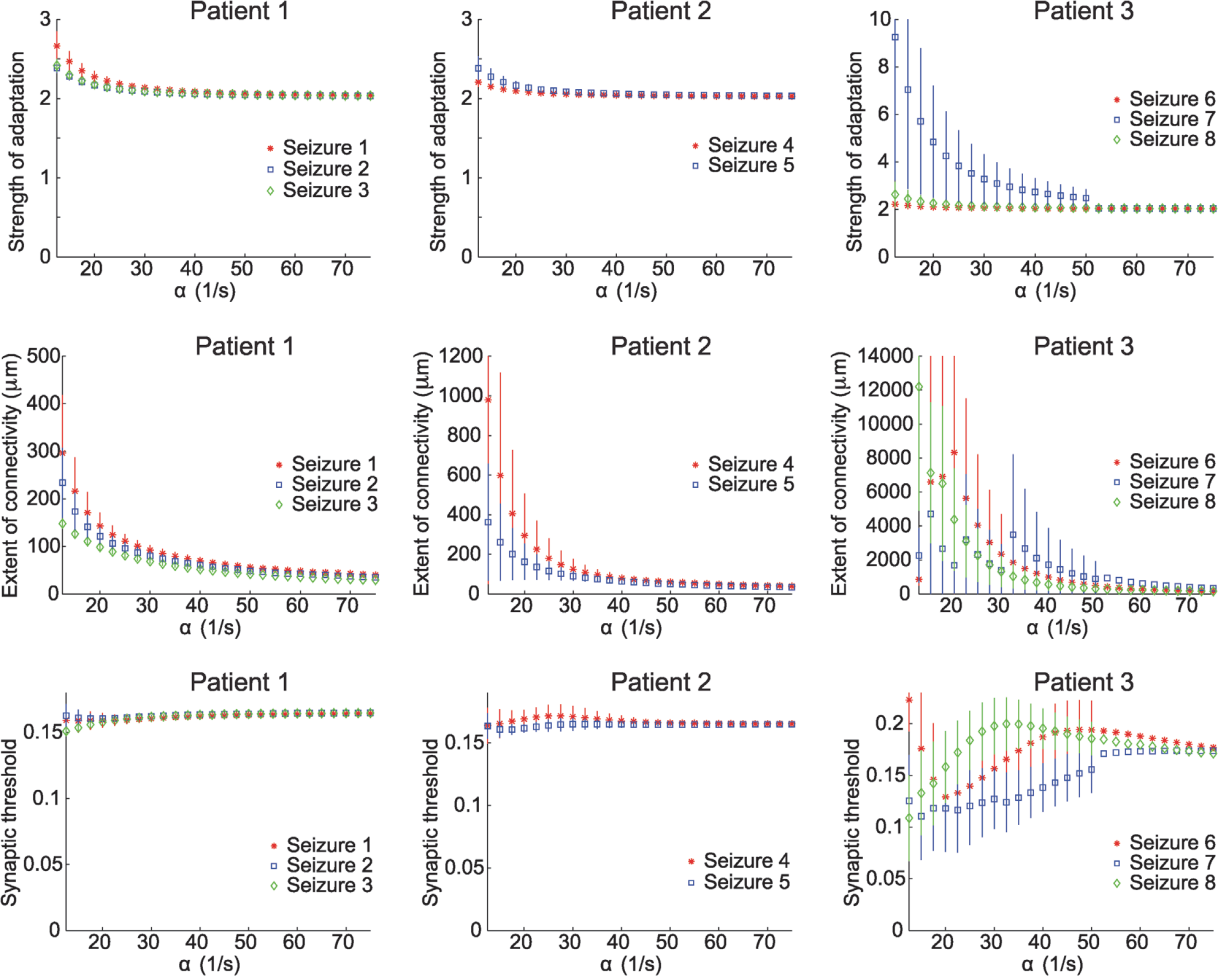
Relationships between the timescale parameter and other model parameters suggest similar features across all patients and seizures. The subplots show the relationship between *α* and *β*_0_ (row 1), α and σ (row 2), and *α* and *k* (row 3). Patient 1 is in column 1, Patient 2 in column 2, and Patient 3 in column 3. For Patient 1, *β*_0_ is between 2 and 3, σ is between 40 and 250 μm/ms, and *k* is between 0.15 and 0.17. For Patient 2, *β*_0_ is between 2 and 3, σ is between 40 and 1000 μm/ms, and *k* is between 0.15 and 0.17. For Patient 3, for 25/s < α <75/s, *β*_0_ is between 2 and 10: σ is between 60 to 4000 μm/ms and *k* is between 0.1 to 0.2.

With the ratio *δ* = *α*/10 now fixed, we proceed to analyze the relationship between *α* and three other model parameters: *β*_0_, *k* and *σ*. We recall that *β*_0_ is the strength of the adaptation and *β*_0_ = *β*/*α*. In Fig. 10 and Table 2 we summarize the results of these relationships for the three patients. Based on the analysis shown in Fig. 9, we examine *α* between 12/s and 75/s, for which the model tends to successfully reproduce the observed waves for all three patients. In particular, above 90% of the analyzed waves are replicated in this range of *α* for all seizures. We find for Patients 1 and 2 that the values of the parameters *β*_0_, *σ*, and *k* tend to remain consistent from seizure to seizure as a function of *α*. We also note that, for *α* sufficiently large (i.e., *α >* 25/s), the variability of these estimates across the traveling wave events is relatively small (Fig. 10). Moreover, the parameter estimates produce similar values, both within each patient and between the two patients (Fig. 10 and Table 2). For Patient 3, we find that the parameter estimates exhibit more dependence on *α* and are more variable. However, even these estimates remain consistent with the other patients and seizures.

**Table 2:** Range of parameters supporting wave propagation, fixing *δ* = α/10.

Seizure number	*α* (1/s)	*β*_0_ (strength)	*σ* (*μ*m)	*k* (synaptic threshold)
1	15–53	2–2.5	50–250	0.15–0.17
2	15–58	2–2.3	40–180	0.16–0.17
3	15–55	2–2.3	40–130	0.15–0.17
4	15–78	2–2.2	40–600	0.16–0.18
5	15–150	2–2.3	20–300	0.16–0.17
6,7, and 8	25–150	2–4	60–4000	0.12–0.2

Seizures 1, 2 and 3 correspond to Patient, 1, Seizures 4 and 5 correspond to Patient 2 and Seizures 6, 7 and 8 correspond to Patient 3. We note that consistent parameter ranges for *β*_0_, *σ* and *k* appear in seizures of the same patient. We also note in comparison with Patient 1, the parameters for *σ* include larger values for Patient 2 and Patient 3.

We note that, for large values of *α*, the estimates of *β*_0_ tend to converge to similar values (Fig. 10). To understand this, we use the explicit formula for *β*_*empirical*_ in terms of the reverberation time: $\beta_{empirical}=\frac{{\left(\delta -\alpha \right)}^{2}}{4\delta }+\frac{4\pi^{2}}{\tau^{2}\delta }=\beta_{max}+\frac{4\pi^{2}}{\tau^{2}\delta }$. Substituting *δ* = *α*/10 we then obtain $\beta_{empirical}=\frac{81}{40}\alpha +\frac{40\pi^{2}}{\tau^{2}\alpha }$. This implies that smaller values of *α* and the reverberation *τ* have bigger impacts on the value of *β*_*empirical*_. Due to the small values of *τ* obtained from Patient 3 (see Table 2), larger variability in the values of *β*_0_ appears at small *α* (Fig. 10). Moreover, since $\beta_{0}=\frac{\beta_{empirical}}{\alpha }=\frac{81}{40}+\frac{40\pi^{2}}{\tau^{2}\alpha^{2}}$, we obtain that as *α* increases *β*_0_ converges to $\frac{81}{40}$ (this limit is determined by the specific choice *δ* = *α*/10), explaining the convergence seen in Fig. 10 to a specific value of *β*_0_. Similar trends appear in the other parameter estimates (Fig. 10) and the implicit equations of the matching conditions determine these trends. To illustrate, we observe in Fig. 7 that as *β* increases (and *β*_0_ decreases), *σ* decreases, explaining the convergence of *σ* as *α* increases (Fig. 10, middle row).

### Numerical simulations of the model produce one-dimensional waves consistent with the LFP data

**Figure 11:**
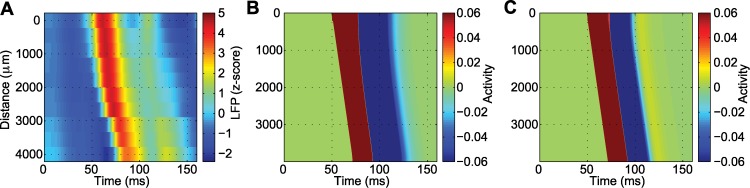
The simulated and observed data are consistent. **(a)** A wave from Seizure 1 with speed *c* = 179 μm/ms and width *w* = 3535 μm. **(b)** Parameters obtained from the real eigenvalue solution, *α* = 7.5/s, *β*_0_ = 2.9, σ = 100 μm. The wave has a speed of *c* = 178 μm/ms and width *w* = 3698 μm. **(c)** Parameters obtained from the complex eigenvalue solution, *α* = 7.5/s, *β*_0_ = 2.5, σ = 160 μm. The wave has a speed of *c* = 178 μm/ms and width *ω* = 3698 μm. The positive activity reverberation in yellow is visible following the main wave in red and blue. The color scale is chosen to allow visualization of the smaller amplitude reverberation.

As a final illustration of the suitability of the model, we consider an example numerical simulation of the model (1) (see Methods). To do so, we choose a particular wave from the LFP data of Seizure 1, estimate *c* and *w* directly from the data, and fix *α* = 7.5/s, as for this value of *α* non-trivial parameters from both the real and complex eigenvalue solutions can be obtained from the matching conditions. Following an initial stimulus (5 ms initial input at position 0 *μ*m) the model produces a traveling pulse that is followed by a smaller amplitude reverberation. A comparison of a wave from the clinical recordings with the real and complex eigenvalues case is shown in Fig. 11. We note that both simulations accurately replicate features of the observed LFP wave (namely, the speed and width), but that the complex eigenvalue case solution also produces a secondary bump of activity consistent with the reverberation in the observed LFP wave. We also note that, in the model, the activity decreases below 0 between the mean crest of the traveling wave and the subsequent reverberation of activity in Fig. 11(c). A decrease in activity also appears in the *in vivo* data between the crest of the traveling wave and the reverberation (example in Fig. 11(a)); however, this decrease is smaller in magnitude than that produced in the model. An updated model that includes inhibition helps reduce this discrepancy, as illustrated in the next subsection.

### Numerical simulations of a model with inhibition produce additional consistency with the LFP data

The original model formulation (1) is analytically tractable and capable of reproducing important features of the observed traveling wave dynamics. However, as expected, this relatively simple model exhibits some inconsistencies with the *in vivo* data, for example the large negativity following the traveling wave crest.

Increasing the complexity of the model through the addition of more biological features may help reduce these inconsistencies. To that end, we consider an updated model that includes an inhibitory population. In particular, we implement the following system:

$$\begin{array}{l} u_{t}\left(x,t\right)=-\alpha_{e}u\left(x,t\right)+\alpha_{e}H\left(\;g_{ee}⊗u(x)-g_{ie}⊗v(x)+P(x,t)-k_{e}\;\right)-\alpha \beta_{0}q\left(x,t\right)\\ q_{t}\left(x,t\right)=\delta u\left(x,t\right)-\delta q\left(x,t\right)\\ v_{t}\left(x,t\right)=-\alpha_{i}v\left(x,t\right)+\alpha_{i}H\left(\;g_{ei}⊗u(x)-g_{ii}⊗v(x)+Q(x,t)-k_{i}\;\right), \end{array}$$ (2)

where *u*(*x*, *t*) is the mean synaptic activity of the excitatory population, *v*(*x*, *t*) is the mean synaptic activity of the inhibitory population, *q*(*x*, *t*) is the adaptation term in the excitatory population, and *P*(*x*, *t*) and *Q*(*x*, *t*) are external inputs to the excitatory and inhibitory populations, respectively. The convolutions account for the spatial extent of the synaptic connectivities,

$$g_{jk}⊗w(x)={\bar{g}}_{jk}\frac{1}{2\sigma_{jk}}\int_{-\infty }^{+\infty }e^{-\frac{|x-y|}{\sigma_{jk}}}w\left(y,t\right)dy,$$

where *j* = {*e*, *i*}, *k* = {*e*, *i*}, and ${\bar{g}}_{jk}=\{0,1\}$. *H* is the Heaviside function, which becomes non-zero when the total input exceeds the threshold *k*_*j*_.

**Figure 12:**
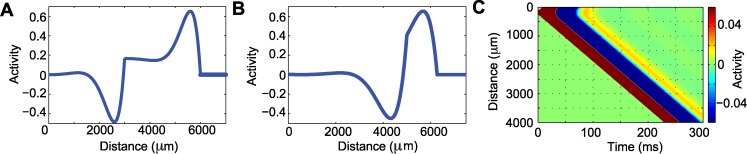
A model that includes inhibition produces additional features consistent with the *in vivo* data. **(a)** Wave profile obtained from the original model without inhibition (${\bar{g}}_{ei}=0$, ${\bar{g}}_{ie}=0$ and ${\bar{g}}_{ii}=0$). The parameters used are *α*_e_ = 25/*s*, *δ* = 2.5/*s*, *β* = 5, σ_ee_ = 52 μm and *k*_e_ = 0.14. The depression of activity reaches approximately -0.5 and is followed by a small reverberation of activity. **(b)** Wave profile obtained in the updated model that includes inhibition (${\bar{g}}_{ei}=1$, ${\bar{g}}_{ie}=1$, ${\bar{g}}_{ii}=1$). The wave has a smoother profile and the depression of activity does not reach -0.5. The width of the wave is reduced to around 1800 μm, in comparison with the 3000 μm of (a). **(c)** Using the parameters of (b), we obtain wave propagation.

To characterize the behavior of this model, we perform numerical simulations. We set the parameters to match the wave speed and width used for the original model (1) in Fig. 5b, and fix *α*_*i*_ = 2.5/s, *k*_*i*_ = 1, *σ*_*ei*_ = 20 *μ*m, *σ*_*ie*_ = 20 *μ*m, and *σ*_*ii*_ = 0. We first consider the case ${\bar{g}}_{ei}=0$, ${\bar{g}}_{ie}=0$ and ${\bar{g}}_{ii}=0$ so that the excitatory and inhibitory populations do not interact. The resulting wave profile (Fig. 12a) reveals a large amplitude pulse, followed by a deep depression of activity, and then a smaller amplitude reverberation, as expected for the original model formulation (1). Then, using the same parameter settings, we activate interactions between the excitatory and inhibitory populations (${\bar{g}}_{ei}=1$, ${\bar{g}}_{ie}=1$, ${\bar{g}}_{ii}=1$). The resulting wave profile (Fig. 12b,c) exhibits qualitative differences from those in the original model; by including inhibition, the wave profile becomes smoother and thinner, and the depression of activity following the large amplitude pulse is shallower. These results suggest that a neural field model with adaptation and inhibition produces wave profiles with additional features consistent with the *in vivo* data, including a smoother wave profile and a shallower depression of activity following the main pulse. We conclude that the original model (1), even in the absence of inhibition, supports wave propagation as observed in the clinical recordings. However, incorporating additional biological features in the model - such as inhibition - may improve fidelity with the clinical data.

## Discussion

In this paper, we considered invasive local field potential (LFP) recordings from a population of human patients during seizures. We showed that, in the late stages of seizures, spatiotemporal patterns of activity propagate across a small patch of cortex. These patterns can be well approximated as one-dimensional plane waves, and we characterized important features of these waves (i.e., the speeds and widths). We found traveling wave speeds of ≈ 80 380 *μ*m/ms, consistent with the propagating velocity of a pulse when GABAergic local inhibition is blocked (e.g., 60–90 *μ*m/ms in [70], 70 *μ*m/ms in [71], 130–190 *μ*m/ms in [25], and 120–150 *μ*m/ms in [72]). In addition, we examined the features of small amplitude “reverberations” in the voltage activity following each wave.

To further characterize the observed LFP waves, we implemented a relatively simple neural field model consisting of an excitatory population of cells with adaptation. This abstract mathematical model is flexible enough to replicate important features of wave propagation near seizure termination for the population of patients and seizures. Moreover, the relative simplicity of the model permits analytic solutions; we showed here, for the first time, that traveling wave solutions exist and are stable in this activity-based model formulation with adaptation. In addition, the model parameters permit biophysical interpretation (e.g., as the extent of synaptic connectivity). By combining analytic model solutions with features of the observed waves - such as the speed and width - we estimated parameters in the model. The estimated parameters included the timescales of activity and adaptation, and the spatial extent of the connectivity. We find that the timescale of the model consistent with the observed LFP data is biologically reasonable: the adaption is an order of magnitude slower than the activity. Measures of synaptic connectivity in a local neighborhood of cortical tissue have been reported to range from 40 *μ*m to 2 mm [12, 41, 63, 73–75]. For the deduced range of parameters obtained in this study, we find that the extent of connectivity, *σ*, for Patients 1 and 2 coincides with this established range. For Patient 3, we obtain connectivities between 60 *μ*m to 4 mm, which is larger, but not wholly inconsistent with existing estimates. We find for all three patients that the parameter *β*_0_, which is the strength of the adaptation, is between 2 and 4; and the parameter k, which accounts for the synaptic threshold, is between 0.12 and 0.2. The variability in the estimates of *σ*, *β*_0_ and *k* may reflect changing biophysical features during seizure (e.g., progressive changes in synaptic efficacy or changes in the extracellular environment) as well as the variability inherent in measuring a noisy biological system. We also note that for the three patients, as the timescale of the activity increases, the extent of the connectivity decreases (Fig. 10) suggesting that faster activities (large *α*) require less distant connectivity. Finally, we note that the parameter estimates are consistent both within individual patients, and across the population of patients and seizures. We conclude from these results the following hypothesis: plane waves observed *in vivo* late in human seizure can be supported in a relatively simple mathematical model without inhibition, consistent with *in vitro* slice and theoretical work (e.g., [25, 36, 70–72, 76–78]). However, we note that inclusion of inhibition may improve features of the model (e.g., may better mimic aspects of the wave profile, see Fig. 12 and S2 Text in Supporting Information for additional illustrations).

The analysis and modeling focused on an interval preceding seizure termination, in which the data have transitioned to large amplitude spike-and-wave (or spike-and-polywave) oscillations. A goal of this modeling study was to simulate some of the spatiotemporal aspects of this spike-and-wave activity. Animal studies suggest the mechanisms that support this spike-and-wave activity are complex. Some studies have suggested that the “wave” component of the spike-and-wave oscillation reflects inhibitory GABAergic processes [79–81]. However, other animal studies instead propose that slow intrinsic currents (e.g., a calcium-activated potassium current) support the “wave” component of the spike-and-wave oscillation [82–87], and *in vitro* slice experiments indicate that features of wave propagation (i.e., wave velocity and wave amplitude) during epileptiform activity do not depend on inhibition [88]. In addition, during seizures with spike-and-wave oscillations, neural populations are (at least transiently) highly active and thereby drive large changes in intra- and extracellular ion concentrations (e.g., intracellular chloride accumulation and extracellular potassium accumulation) [89]. This may result in pathological changes in brain dynamics, for example the reversal potential of GABA-receptor-mediated inhibitory postsynaptic potentials may shift to positive values [85], and inhibitory mechanisms may engage in the generation of the depolarizing component of spike-and-wave oscillation.

Here we have implemented a mathematical model with a tight focus on one aspect of the late seizure interval: the (approximately) one-dimensional traveling waves that appear in spike-and-wave oscillations near seizure termination. In doing so, we have presented a modeling formulation more consistent with the proposed intrinsic current mechanisms of spike-and-wave oscillations. Nevertheless, we suspect that inhibition plays a fundamental role in seizure, for example at seizure onset [90, 91] when fast-spiking interneurons are highly active. We expect that the addition of more biophysical features to the model (including inhibition) will permit a better match to the *in vivo* LFP data (see Fig. 12 and S2 Text of Supporting Information), at the cost of increased model complexity and reduced analytic tractability.

In this work we implemented a relatively simple one-dimensional neural population model, consisting of a synaptic activity variable and an adaptation variable. The simplicity of the model allows rigorous mathematical analysis, although the biophysical mechanisms remain relatively abstract. The validity of the model is based on the reproduction of wave features present near seizure termination, and parameter estimates consistent with known physiology (i.e., estimates of synaptic connectivity and difference in timescales). The purpose of this model is not to capture the detailed biophysical mechanisms of seizure, as in more realistic computational models [92, 93]. However, we may use the mathematical model to make the following prediction: the traveling waves near seizure termination represent relatively “simple” brain phenomena. Consistent with this notion, we hypothesize that the diversity of complex components that support normal cortical function (e.g., the diversity of inhibitory neuronal populations [94, 95]) have shut down, and allowed these simple dynamics to dominate. Restoration of this diversity and complexity (e.g., activation of silenced inhibitory neuronal populations) would then help disrupt these pathologically organized and simple traveling waves.

To further validate the model results, *in vitro* experiments that reproduce important features of the human *in vivo* data (e.g., the spectrographic properties [90, 96]) would allow detailed pharmacological exploration of the proposed biophysical mechanism of this model. In particular, the more abstract model parameters (like *β*_0_, the strength of the adaptation) may be better understood in terms of specific neuronal mechanisms through experiments in controlled biological systems. These experiments may in turn motivate future work developing more biologically detailed models to provide additional insight into the spatiotemporal dynamics of seizure activity. One important future modeling direction is the further analysis and inclusion of inhibitory populations in this activity-based formulation. Such inclusions may further illuminate the mechanisms of wave propagation, and might help to explain differences in waves seen during the initial and terminal stages of human seizure.

We have focused here on the analysis of the observed LFP plane waves near seizure termination. Rich spatiotemporal patterns also emerged in the clinical LFP data throughout the seizure (and perhaps in other functional states, such as sleep) and will require an expanded two-dimensional model for characterization. For example, we note that near seizure onset complex spatiotemporal patterns emerge, without obvious traveling wave dynamics. The mechanisms that govern the transition from these disorganized spatiotemporal dynamics to more organized traveling waves remain unknown. The analysis of seizures from more patients may help to develop more sophisticated - and biologically detailed models - to explain these complex phenomena. The combination of quantitative data analysis and mathematical modeling of seizure activity across space remains an active research area with important implications for improved treatment of epilepsy.

## Materials and Methods

### Ethics Statement

All patients were enrolled after informed consent was obtained and approval was granted for these studies by local Institutional Review Boards.

### Data Analysis

For each patient and seizure, we analyzed a subset of the diverse spatiotemporal patterns observed approaching seizure termination. We focus here on the analysis of one-dimensional plane waves of activity, which were the most common type of wave we observed in Patients 1 and 2 (Seizure 1, 36 out of 40 waves; Seizure 2, 36 out of 41; Seizure 3, 39 out of 59; Seizure 4, 26 out of 33; Seizure 5, 35 out of 52). Upon visual inspection, the excluded waves exhibited different spatiotemporal patterns, including disorganized waves of high activity, and two-dimensional patterns, such as waves that initiated at the center of the microelectrode array, and spiral waves. Again, we focus here only on the one-dimensional plane waves and estimates of the model parameters from these waves. For Patient 3, we focused on a contiguous half (2 mm by 4 mm) subsection of the entire (4 mm by 4 mm) microelectrode array. For this patient, we were able to detect waves moving closer to the horizontal direction (from −45° to 45° and from 135° to 225°). Having selected these one-dimensional waves from the three patients, all waves were analyzed using the same set of data analysis algorithms described below. Components of these data may be made available by request to the corresponding author.

**Figure 13:**
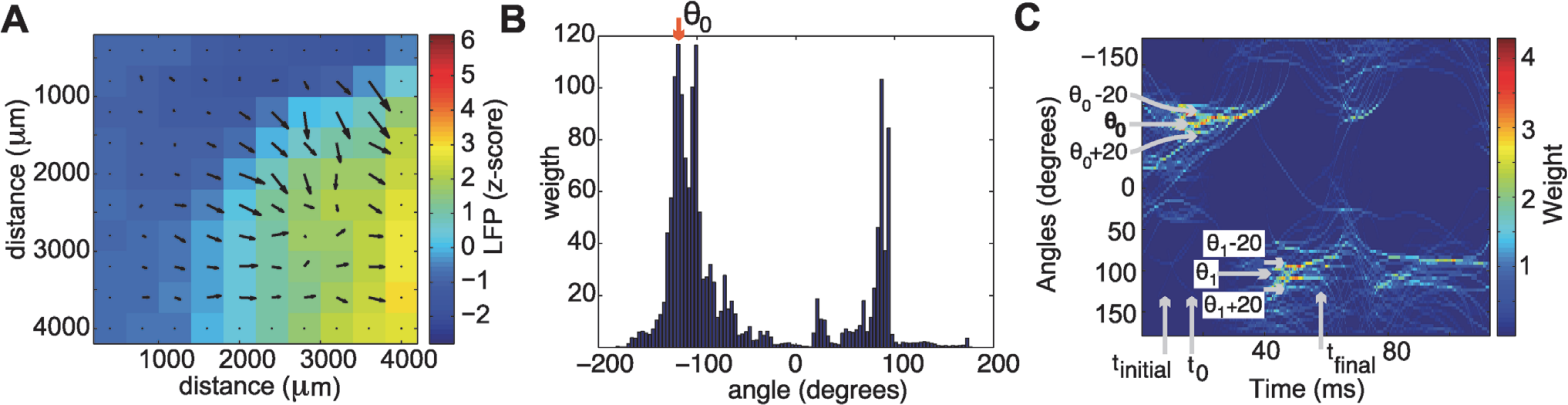
Example of LFP data analysis procedure. **(a)** Example of a vector field before a wave enters the microelectrode array. For each of the interior electrodes, an angle is assigned according to the gradient. **(b)** Example weighted angle distribution for the time interval (*t*_0_ − 14 ms, *t*_0_ + 100 ms) for a single wave during a seizure. In this example, *t*_*initial*_ = 14 ms and *t*_final_ = 100 ms. The peak of the distribution occurs at angle *θ*_0_. **(c)** Illustration of the different computed quantities: *θ*_0_ is the peak of the distribution; *θ*_1_ = *θ*_0_+180*c*, where *c* = ±1 (depending on the value of *θ*_0_); *t*_*initial*_ is the time at which the phase interval (*θ*_0_-20, *θ*_0_+20) acquires non-zero counts; *t*_0_ is the time at which the LFP z-scored signal at the center of the microelectrode array exceeds a threshold; and *t*_final_ is the last time at which counts appear in the angular interval around *θ*_1_.

The purposes of the data analysis were: i) To obtain a time interval for the propagation of each planar wave; ii) To obtain the direction of wave propagation; iii) To obtain the different one-dimensional paths through the two-dimensional microelectrode array for a given direction; iv) To obtain the speed, width, and reverberation time along each one-dimensional path; and v) To obtain the mean speed, mean width and mean reverberation time for each wave across different paths. To determine the time interval for the propagation of each planar wave, we computed the gradient of the LFP activity at each moment in time. The gradient assigns to each spatial location a vector specifying the direction and magnitude of maximal increase in activity (Fig. 13a). To compute the gradient, we analyzed voltage differences between adjacent electrodes. A histogram of the angles of the gradient at each position, weighted by the magnitude of the gradient, was then constructed for each moment in time (Fig. 13b). We label *t*_0_ the time at which the LFP z-scored signal at the center of the microelectrode array exceeded a threshold of 2.5. We then determined the peak of the unimodal angle distribution at time *t*_0_, which we labeled *θ*_0_. We considered angles between *θ*_0_-20 and *θ*_0_+20 degrees and analyzed the proportion of angles within the interval (*θ*_0_-20, *θ*_0_+20), forward and backwards in time starting at *t*_0_. The time *t*_*initial*_ denotes the first time at which the number of counts in the angular interval becomes non-zero. The time *t*_final_ is the last time at which counts appear in the angular interval. In this way, each wave is assigned a time interval (*t*_*initial*_, *t*_*final*_) for which angles appear in the interval (*θ*_0_-20, *θ*_0_+20). In this time interval, the weighted histograms of the angles showed a clear organization of the gradient directions and appearance of two peaks in the histogram distributions (Fig. 13b). These two peaks account for the preferred angle before the wave enters the microelectrode array and after the wave exits the microelectrode array. To determine the direction of each wave we focused on the first peak (Fig. 13b). This peak typically occurs in the time interval (*t*_*initial*_, *t*_0_). In addition, we visually inspected each peak and verified that the associated angle accurately described the direction of propagation for each wave. The notions of *t*_0_, *t*_*initial*_, *t*_*final*_ and *θ*_0_ are illustrated in Fig. 13c.

**Figure 14:**
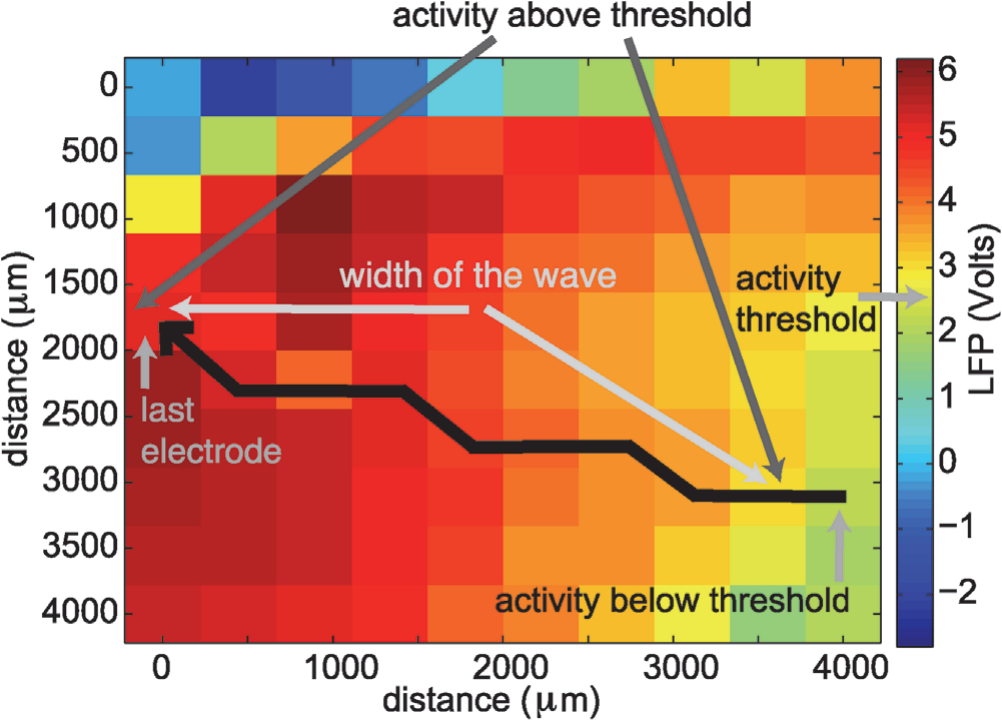
The width of a wave along a one-dimensional path is defined as the distance from the “last electrode” to the first electrode along the path whose activity is below the activity threshold.

**Figure 15:**
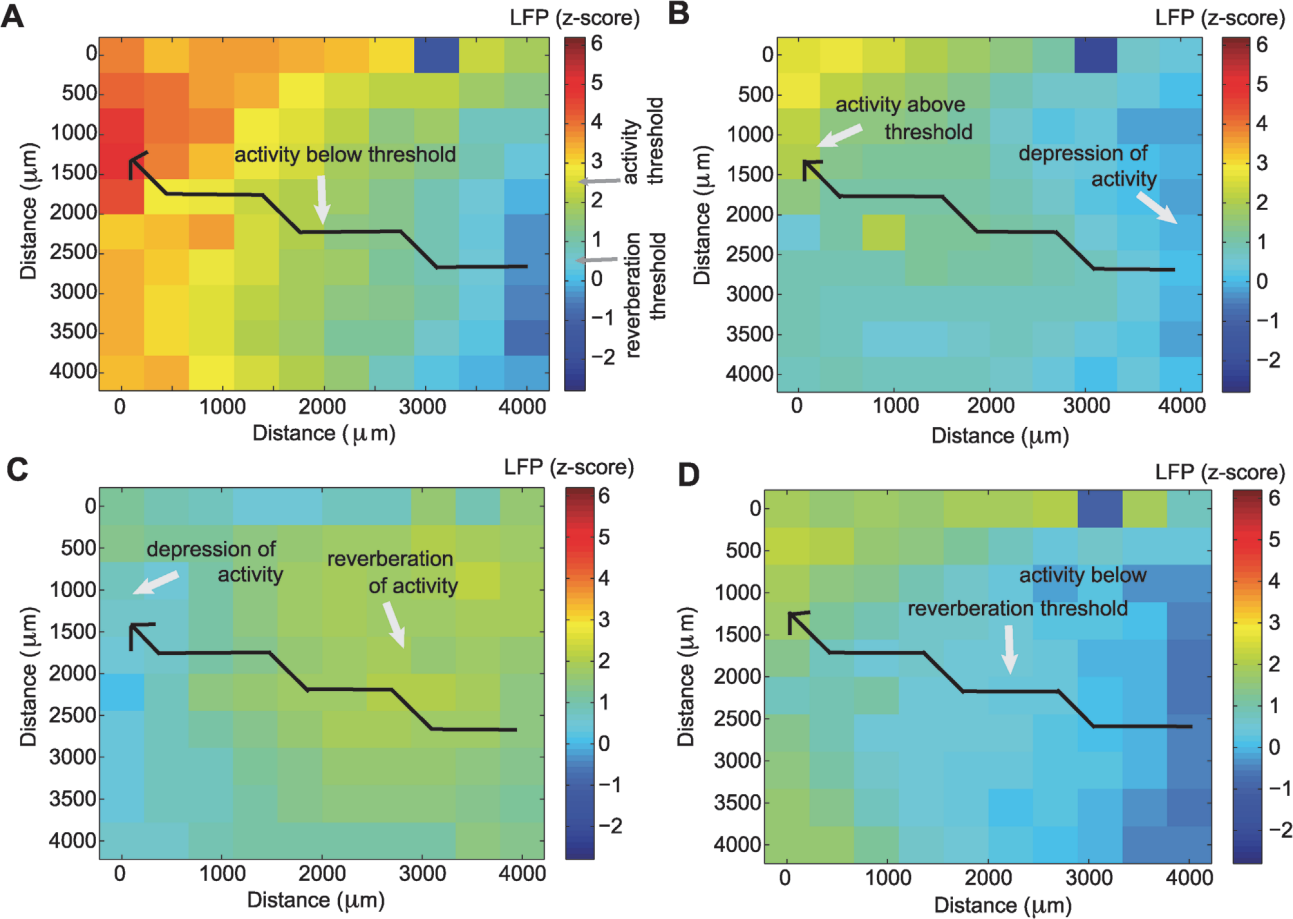
Illustration of the measurement of the “reverberation” time. **(a)** For each electrode along a one-dimensional path we compute the time at which the activity decreases below an activity threshold (marked in the LFP colorbar) after a wave of large amplitude activity. **(b)** A depression of activity follows a wave of high activity. **(c)** A reverberation of activity follows the depressed state. **(d)** We compute the reverberation time as the duration between the activity at the center of the path decreasing below an activity threshold (a), and then the activity first increasing above - and then receding below - a reverberation threshold (marked in the LFP colorbar). We compute this time difference for each of the electrodes along the one-dimensional paths.

Having determined the angle at which LFP activity propagated, we then constructed one-dimensional paths spanning the microelectrode array. Each path consisted of 10 adjacent electrodes and ran parallel to the direction of the observed wave. Along each such path we determined the speed and width of the wave. For each path, we determined the time at which the activity at each electrode exceeded a threshold of one standard deviation above the mean LFP computed for the entire duration of seizure termination investigated. In this way, every electrode along a path was assigned a time of wave onset, which was used to compute the speed. We used all possible combinations of the 10 electrodes along each one-dimensional path to compute the speed, resulting in a total of 45 estimates of speed. To mitigate the impact of outliers, the speed for each one-dimensional path was then calculated as the median of the 45 speed estimates. We then estimated the speed for each wave as the mean speed among the different one-dimensional paths. Depending on the direction of the wave, from the 10 electrodes that form a one-dimensional path, there is one electrode at which the large amplitude activity of the wave reaches last, and we label this the “last electrode” (example in Fig. 14). To measure the wave width, for each one-dimensional path we computed the time at which the activity at the last electrode exceeded a threshold of 2.5 of the LFP z-scored signal. At that instant in time, the activity of the other electrodes along the path was also determined. The location at which the activity transitioned from above the threshold (of 2.5 of the LFP z-scored signal) to below the threshold was determined. The spatial extent from the last electrode to this transition point on the one-dimensional path defined the width of the wave. An illustration of the wave width determination is shown in Fig. 14. We note that if no electrode along the one-dimensional path transitioned to below the threshold, then the wave covered the entire spatial extent of the path, and the width of the wave indicates a lower bound. For each wave, the width refers to the mean widths obtained from all one-dimensional paths. To obtain the reverberation time we first determined the time at which the large amplitude wave of activity fell below a threshold of 0.5 of the LFP z-scored signal; we consider this time as the “end” of the primary traveling wave. Starting from this time point, we then determined the time for the activity to first exceed a reverberation threshold, defined as 0.5 of the LFP z-scored signal, and then for the activity to decrease again below this threshold. This decrease below the reverberation threshold defined the reverberation time. For an illustration of the reverberation time, see Fig. 15. We computed the reverberation time for each electrode along the one-dimensional path. The mean among the different one-dimensional paths gave the reverberation time of each wave. Using a t-test for small samples we computed a 90% confidence interval for the mean speed and mean width of each wave (Fig. 3), where the number of samples was given by the number of one-dimensional paths existent for each wave.

### Mathematical Model

In the section, we describe in detail the mathematical analysis of the model (1). We note that the model (1) supports traveling front solutions when the adaptation term is removed. However, these front solutions are not consistent with observed LFP activity, and therefore not examined here.

As mentioned in Results, we use the moving frame *z* = *x*-*ct* and identify stationary solutions in this frame. These solutions will be of the form *u*(*x*, *t*) = *u*(*x*-*ct*, *t*) = *u*(*z*, *t*) and *q*(*x*, *t*) = *q*(*x*-*ct*, *t*) = *q*(*z*, *t*), such that *u*_*t*_(*z*, *t*) = 0 and *q*_*t*_(*z*, *t*) = 0. We use the connectivity function $w(z)=\frac{1}{2\sigma }e^{-\frac{\left|z\right|}{\sigma }}$. By making this change of variables, we obtain the system of differential-integral equations

$$\begin{array}{l} -cu^{\prime }\left(z\right)=-\alpha u\left(z\right)+\alpha H\left(\int_{-\infty }^{\infty }w(\bar{z}-z)u\left(\bar{z}\right)d\bar{z}-k\right)-\beta q\left(z\right)\\ -cq^{\prime }\left(z\right)=\;\;\;\;\delta u\left(z\right)-\delta q\left(z\right), \end{array}$$

which can be rewritten in the form

$$\left(\begin{array}{c} u^{\prime }(z)\\ q^{\prime }(z) \end{array}\right)=\left(\begin{array}{cc} \alpha /c & \beta /c\\ -\delta /c & \delta /c \end{array}\right)\left(\begin{array}{c} u(z)\\ q(z) \end{array}\right)+\left(\begin{array}{c} -\frac{\alpha }{c}H\left(\int_{-\infty }^{\infty }w(\bar{z}-z)u\left(\bar{z}\right)d\bar{z}-k\right)\\ 0 \end{array}\right).$$ (3)

We assume *c* *>* 0 which corresponds to a rightward moving wave. An analogous consideration holds for leftward moving waves (*c* *<* 0). We note that the nonlinear part of system (3) will be either zero or nonzero depending on the Heaviside function. For that reason the system can be analyzed by considering when the Heaviside function is zero (Case 1), and when the Heaviside function is non-zero (Case 2). We consider both cases below.

#### Case 1. Heaviside function is zero

This occurs when $\int_{-\infty }^{\infty }w(\bar{z}-z)u\left(\bar{z}\right)d\bar{z}<k$. In this case we obtain the following linear system:

$$\left(\begin{array}{c} u^{\prime }(z)\\ q^{\prime }(z) \end{array}\right)=\left(\begin{array}{cc} \alpha /c & \beta /c\\ -\delta /c & \delta /c \end{array}\right)\left(\begin{array}{c} u(z)\\ q(z) \end{array}\right).$$ (4)

Depending on the parameters of the model, we will obtain real eigenvalues or complex eigenvalues for the system (4). We consider each scenario in turn:

#### Real eigenvalues when the Heaviside function is zero

This scenario occurs when $\beta <\frac{{\left(\delta -\alpha \right)}^{2}}{4\delta }$. Solving for the eigenvalues of system (4) we find,

$$\lambda_{\pm }=\frac{1}{2c}\left(\left(\delta +\alpha \right)\pm \sqrt{{\left(\delta +\alpha \right)}^{2}-4\delta \left(\alpha +\beta \right)}\right)$$

with corresponding eigenvectors

$$V_{\pm }=\left(\begin{array}{c} \delta -\alpha \mp \sqrt{{\left(\delta +\alpha \right)}^{2}-4\delta \left(\alpha +\beta \right)}\\ 2\delta \end{array}\right).$$

Consider

$$U(z)=\left(\begin{array}{c} u(z)\\ q(z) \end{array}\right).$$

Then the solution of system (4) with real eigenvalues will have the form *U*(*z*) = *a*_1_ *V*_+_ *e* ^λ+z^ + *a*_2_ *V*_*e* ^λ_z^. We note that λ_±_ ≥ 0.

#### Complex eigenvalues when the Heaviside function is zero

This scenario occurs when $\beta >\frac{{\left(\delta -\alpha \right)}^{2}}{4\delta }$. The complex eigenvalues are given by

$$\lambda_{\pm }=\frac{1}{2c}\left[\left(\delta +\alpha \right)\pm i\sqrt{4\delta \left(\alpha +\beta \right)-{\left(\delta +\alpha \right)}^{2}}\right].$$

It is only necessary to solve for the eigenvector associated with λ_+_ to obtain the general solution of the linear system. Solving for the complex eigenvector associated with λ_+_ we find:

$$V_{+}=\left(\begin{array}{c} 2\beta \\ \delta -\alpha \end{array}\right)+i\left(\begin{array}{c} 0\\ \sqrt{4\delta \left(\alpha +\beta \right)-{\left(\delta +\alpha \right)}^{2}} \end{array}\right).$$

With this eigenvector we can obtain the imaginary solution. This solution can be simplified and we can obtain from it two linearly independent eigenvectors V_1_ and V_2_. To simplify the computations we do not show this term and instead focus on constructing the traveling wave solution.

#### Case 2. Heaviside function is nonzero

This scenario occurs when $\int_{-\infty }^{\infty }w(\bar{z}-z)u\left(\bar{z}\right)d\bar{z}>k$. In this case, system (3) simplifies to:

$$\left(\begin{array}{c} u^{\prime }(z)\\ q^{\prime }(z) \end{array}\right)=\left(\begin{array}{cc} \alpha /c & \beta /c\\ -\delta /c & \delta /c \end{array}\right)\left(\begin{array}{c} u(z)\\ q(z) \end{array}\right)+\left(\begin{array}{c} -\alpha /c\\ 0 \end{array}\right).$$ (5)

We note that the analysis of system (5) applies to the homogeneous part of (4). In particular we obtain the same eigenvalues (either purely real or complex) and eigenvectors. We only need to obtain a particular solution for the inhomogeneous part of the system in order to obtain the complete solution of (5). A particular solution of the inhomogeneous system is:

$$U_{p}=\left(\begin{array}{c} \frac{\alpha }{\alpha +\beta }\\ \frac{\alpha }{\alpha +\beta } \end{array}\right).$$

#### Traveling Wave Solutions

Our goal is to obtain a traveling wave solution using the properties we found for the two cases (Case 1 and Case 2) described above. Special care must be taken at the point where the system changes from Case 1 to Case 2. Biologically, this corresponds to the point where the system passes through the synaptic threshold. In looking for a wave-type solution, we assume that the input from the activity crosses a threshold *k* two times. We also assume that the traveling wave solution is continuous. Using these assumptions we are able to solve for all of the unknown coefficients.

#### Traveling Wave Solution - Real Eigenvalues

Using the results for the two cases of the Heaviside function (Case 1 and Case 2, described above), we now focus on establishing a traveling wave solution of (1). In particular, we look for a traveling pulse that transitions from a state of rest to an excited state, and then returns to rest. To do so, the interaction term must cross the synaptic threshold *k* at exactly two points. For simplicity, and without loss of generality, we assume that these points are 0 and *w*, where *w* is the width of the wave. We then obtain a traveling wave solution of the form:

$$U(z)=\{\begin{array}{ll} a_{1}V_{+}e^{\lambda_{+}z}+a_{2}V_{-}e^{\lambda_{-}z} & \text{if}z\geq w\\ a_{3}V_{+}e^{\lambda_{+}z}+a_{4}V_{-}e^{\lambda_{-}z}+\left(\begin{array}{c} \frac{\alpha }{\alpha +\beta }\\ \frac{\alpha }{\alpha +\beta } \end{array}\right) & \text{if}0<z<w\\ a_{5}V_{+}e^{\lambda_{+}z}+a_{6}V_{-}e^{\lambda_{-}z} & \text{if}z\leq 0. \end{array}$$

We use the condition *u*(*z*) → 0 as *z* → ∞ to determine that activity *u*(*z*) = 0 for *z* ≥ *w*. Also, by the assumption that the traveling wave solutions are continuous, and the assumption that the wave passes through the threshold at *z* = 0 and *z* = *w*, we can solve for the rest of the unknown coefficients in the above system of equations and obtain the traveling wave solution of the activity in the real eigenvalue case:

$$u(z)=\{\begin{array}{ll} 0 & \text{if}z\geq w\\ \frac{\alpha }{\delta \left(\alpha +\beta \right)\left(\lambda_{+}-\lambda_{-}\right)}\left[\lambda_{-}e^{\lambda_{+}\left(z-w\right)}\left(\delta -c\lambda_{+}\right)-\lambda_{+}e^{\lambda_{-}\left(z-w\right)}\left(\delta -c\lambda_{-}\right)+\delta \left(\lambda_{+}-\lambda_{-}\right)\right] & \\ & \text{if}0<z<w\\ \frac{\alpha }{\delta \left(\alpha +\beta \right)\left(\lambda_{+}-\lambda_{-}\right)}\left[\lambda_{-}\left(e^{-w\lambda_{+}}-1\right)\left(\delta -c\lambda_{+}\right)e^{\lambda_{+}z}+\lambda_{+}\left(1-e^{-w\lambda_{-}}\right)\left(\delta -c\lambda_{-}\right)e^{\lambda_{-}z}\right] & \\ & \text{if}z\leq 0 \end{array}$$

where $\lambda_{\pm }=\frac{1}{2c}\left[\alpha +\delta \pm \sqrt{{\left(\alpha +\delta \right)}^{2}-4\delta (\alpha +\beta )}\right]$.

#### Traveling Wave Solution - Complex Eigenvalues

The analysis of the traveling wave solution in the complex eigenvalue case is similar to the real eigenvalue case, we find that the traveling wave solution has the form:

$$U(z)=\{\begin{array}{ll} \exp(\frac{\alpha +\delta }{2c}z)\left[b_{1}V_{1}+b_{2}V_{2}\right] & \text{if}z\leq 0\\ \exp(\frac{\alpha +\delta }{2c}z)\left[b_{3}V_{1}+b_{4}V_{2}\right]+\left(\begin{array}{c} \frac{\alpha }{\alpha +\beta }\\ \frac{\alpha }{\alpha +\beta } \end{array}\right) & \text{if}0<z<w\\ 0 & \text{if}z\geq w. \end{array}$$

By assuming that the solution at the points 0 and *w* is continuous, we deduce two systems of equations that can be solved to obtain corresponding coefficients. The resulting traveling wave solution for the activity is:

$$u(z)=\{\begin{array}{ll} 0 & \text{if}z\geq w\\ \frac{\alpha \exp(\frac{\alpha +\delta }{2c}(z-w))}{\sqrt{{\left(\delta +\alpha \right)}^{2}-4\delta \left(\alpha +\beta \right)}\left(\alpha +\beta \right)}\left[c_{3}\;\text{cos}(z\frac{\sqrt{4\delta \left(\alpha +\beta \right)-{\left(\delta +\alpha \right)}^{2}}}{2c})+c_{4}\;\text{sin}(z\frac{\sqrt{4\delta \left(\alpha +\beta \right)-{\left(\delta +\alpha \right)}^{2}}}{2c})\right]+\frac{\alpha }{\alpha +\beta } & \\ & \text{if}0<z<w\\ \frac{\alpha \exp(\frac{\alpha +\delta }{2c}z)}{\sqrt{{\left(\delta +\alpha \right)}^{2}-4\delta \left(\alpha +\beta \right)}\left(\alpha +\beta \right)}\left[c_{1}\;\text{cos}(z\frac{\sqrt{4\delta \left(\alpha +\beta \right)-{\left(\delta +\alpha \right)}^{2}}}{2c})+c_{2\;}\text{sin}(z\frac{\sqrt{4\delta \left(\alpha +\beta \right)-{\left(\delta +\alpha \right)}^{2}}}{2c})\right] & \\ & \text{if}z\leq 0 \end{array}$$

where

$$\begin{array}{l} c_{1}=\sqrt{4\delta \left(\alpha +\beta \right)-{\left(\delta +\alpha \right)}^{2}}+\text{exp}(-w\frac{\alpha +\delta }{2c})\left[\left(2\beta +\alpha -\delta \right)\;\text{sin}(w\frac{\sqrt{4\delta \left(\alpha +\beta \right)-{\left(\delta +\alpha \right)}^{2}}}{2c})-\sqrt{4\delta \left(\alpha +\beta \right)-{\left(\delta +\alpha \right)}^{2}}\;\text{cos}(w\frac{\sqrt{4\delta \left(\alpha +\beta \right)-{\left(\delta +\alpha \right)}^{2}}}{2c})\right]\\ c_{2}=\left(2\beta +\alpha -\delta \right)+\text{exp}(-w\frac{\alpha +\delta }{2c})\left[-\left(2\beta +\alpha -\delta \right)\;\;\text{cos}(w\frac{\sqrt{4\delta \left(\alpha +\beta \right)-{\left(\delta +\alpha \right)}^{2}}}{2c})-\sqrt{4\delta \left(\alpha +\beta \right)-{\left(\delta +\alpha \right)}^{2}}\;\text{sin}(w\frac{\sqrt{4\delta \left(\alpha +\beta \right)-{\left(\delta +\alpha \right)}^{2}}}{2c})\right]\\ c_{3}=\left(2\beta +\alpha -\delta \right)\;\text{sin}(w\frac{\sqrt{4\delta \left(\alpha +\beta \right)-{\left(\delta +\alpha \right)}^{2}}}{2c})-\sqrt{4\delta \left(\alpha +\beta \right)-{\left(\delta +\alpha \right)}^{2}}\;\text{cos}(w\frac{\sqrt{4\delta \left(\alpha +\beta \right)-{\left(\delta +\alpha \right)}^{2}}}{2c})\\ c_{4}=-\left(2\beta +\alpha -\delta \right)\;\text{cos}(w\frac{\sqrt{4\delta \left(\alpha +\beta \right)-{\left(\delta +\alpha \right)}^{2}}}{2c})-\sqrt{4\delta \left(\alpha +\beta \right)-{\left(\delta +\alpha \right)}^{2}}\;\text{sin}(w\frac{\sqrt{4\delta \left(\alpha +\beta \right)-{\left(\delta +\alpha \right)}^{2}}}{2c}) \end{array}$$

Recall the identity $a\;\text{sin}\;x+b\;\text{cos}\;x=\sqrt{a^{2}+b^{2}}\;\text{sin}\;x+\phi$, where

$$\phi =\tan^{-1}\frac{b}{a}+\{\begin{array}{ll} 0 & \text{if}a\geq 0\\ \pi & \text{if}a<0. \end{array}$$

Using the previous identity we can further simplify the traveling wave solution to obtain:

$$u(z)=\{\begin{array}{ll} 0 & \text{if}z\geq w\\ \frac{\alpha }{\alpha +\beta }+\frac{2\alpha \sqrt{\beta }\;\text{exp}(\frac{\alpha +\delta }{2c}(z-w))}{\sqrt{\left(\alpha +\beta \right)\left(4\beta \delta -{\left(\delta -\alpha \right)}^{2}\right)}}\;\text{sin}(\frac{\sqrt{4\delta \beta -{\left(\alpha -\delta \right)}^{2}}}{2c}z+\phi_{1}) & \text{if}0<z<w\\ \frac{2\alpha \sqrt{\beta }\;\text{exp}(\frac{\alpha +\delta }{2c}z)}{\sqrt{\left(\alpha +\beta \right)\left(4\beta \delta -{\left(\delta -\alpha \right)}^{2}\right)}}\;D\;\text{cos}(\frac{\sqrt{4\delta \beta -{\left(\alpha -\delta \right)}^{2}}}{2c}z+\phi_{2}) & \text{if}z\leq 0, \end{array}$$

where

$D=\sqrt{1-2\;\text{exp}(-w\frac{\alpha +\delta }{2c})\;\text{cos}(\frac{\sqrt{4\delta \beta -{\left(\alpha -\delta \right)}^{2}}}{2c}w)+\text{exp}(-w\frac{\alpha +\delta }{c})}$
$\phi_{1}={\text{tan}}^{-1}(\frac{A_{1}}{A_{2}})+\{\begin{array}{ll} \pi & \text{if}A_{1}<0\\ 0 & \text{if}A_{1}>0 \end{array}$
$\phi_{2}={\text{tan}}^{-1}(\frac{A_{3}}{A_{4}})+\{\begin{array}{ll} \pi & \text{if}A_{4}<0\\ 0 & \text{if}A_{4}>0 \end{array}$
$A_{1}=\left(2\beta +\alpha -\delta \right)\;\text{sin}(\frac{\sqrt{4\delta \beta -{\left(\alpha -\delta \right)}^{2}}}{2c}w)-\sqrt{4\delta \beta -{\left(\delta -\alpha \right)}^{2}}\;\text{cos}(\frac{\sqrt{4\delta \beta -{\left(\alpha -\delta \right)}^{2}}}{2c}w)$
$A_{2}=-\left(2\beta +\alpha -\delta \right)\;\text{cos}(\frac{\sqrt{4\delta \beta -{\left(\alpha -\delta \right)}^{2}}}{2c}w)-\sqrt{4\delta \beta -{\left(\delta -\alpha \right)}^{2}}\;\text{sin}(\frac{\sqrt{4\delta \beta -{\left(\alpha -\delta \right)}^{2}}}{2c}w)$
$A_{3}=\sqrt{4\delta \beta -{\left(\alpha -\delta \right)}^{2}}+\;\text{exp}(-\frac{\alpha +\delta }{2c}w)A_{1}$
$A_{4}=(2\beta +\alpha -\delta )+\;\text{exp}(-\frac{\alpha +\delta }{2c}w)A_{2}.$

We note that the period of the damped solution is $(4\pi )/\sqrt{4\delta \beta -{\left(\alpha -\delta \right)}^{2}}$.

### Matching Conditions

In order to ensure the continuity of the solutions, we look at the change points from Case 1 to Case 2. In particular, $k=\frac{1}{2\sigma }\int_{-\infty }^{+\infty }e^{-\frac{|x-y|}{\sigma }}u\left(y,t\right)dy$ at the points *x* = 0 and *x* = *w*. This assumption gives rise to the matching conditions. Once the explicit traveling wave solutions are obtained, it is possible to solve for the exact value of the threshold *k* given by the matching conditions. We list below the solutions for the matching conditions in the case of real eigenvalues and complex eigenvalues.

#### Matching Conditions: Real Eigenvalues

The matching conditions at points 0 and *w* for the real eigenvalue case are:

$$\begin{array}{l} k_{1}=\frac{1}{2\sigma }\int_{-\infty }^{+\infty }e^{-\frac{|y|}{\sigma }}u\left(y,t\right)dy\\ k_{1}=\frac{\alpha \lambda_{-}\lambda_{+}\sigma \;\text{exp}(-\frac{w}{\sigma })\left(\text{exp}(\frac{w}{\sigma })-1\right)(c+\delta \sigma )}{2\delta (\alpha +\beta )\left(\lambda_{-}\sigma +1\right)\left(\lambda_{+}\sigma +1\right)} \end{array}$$

and

$$\begin{array}{c} k_{2}=\frac{1}{2\sigma }\int_{-\infty }^{\infty }e^{-\frac{|y-w|}{\sigma }}u\left(y,t\right)dy\\ k_{2}=\frac{\alpha }{2\delta \left(\lambda_{+}-\lambda_{-}\right)\left(\alpha +\beta \right)}\left[\frac{\lambda_{+}(\delta -c\lambda_{-})}{e^{\lambda_{-}w}\left(\lambda_{-}\sigma -1\right)}+\frac{\lambda_{-}\left(\lambda_{-}-\lambda_{+}\right)\lambda_{+}\sigma (\delta \sigma -c)}{\left(\lambda_{-}\sigma -1\right)\left(\lambda_{+}\sigma -1\right)e^{\frac{w}{\sigma }}}\right]\\ -\frac{\alpha }{2\delta \left(\lambda_{+}-\lambda_{-}\right)\left(\alpha +\beta \right)}\left[\frac{\lambda_{-}\left[c\lambda_{+}\left(-\lambda_{+}\sigma e^{\lambda_{+}w}\left(e^{\lambda_{-}w}-1\right)-e^{\lambda_{-}w}+e^{\lambda_{+}w}\right)-\delta e^{\lambda_{-}w}\left(e^{\lambda_{+}w}-1\right)\right]}{e^{\left(\lambda_{-}+\lambda_{+}\right)w}\left(\lambda_{-}\sigma +1\right)\left(\lambda_{+}\sigma +1\right)}\right]\\ -\frac{\alpha }{2\delta \left(\lambda_{+}-\lambda_{-}\right)\left(\alpha +\beta \right)}\left[\frac{\lambda_{-}^{2}\sigma e^{\lambda_{-}w}\left(e^{\lambda_{+}w}-1\right)\left(\delta -c\lambda_{+}\right)+\delta \lambda_{+}\left(e^{\lambda_{-}w}-1\right)e^{\lambda_{+}w}\left(\lambda_{+}\sigma +1\right)}{e^{\left(\lambda_{-}+\lambda_{+}\right)w}\left(\lambda_{-}\sigma +1\right)\left(\lambda_{+}\sigma +1\right)}\right]\\ -\frac{\alpha }{2\delta \left(\lambda_{+}-\lambda_{-}\right)\left(\alpha +\beta \right)}\left[\frac{\lambda_{-}\left(\delta -c\lambda_{+}\right)}{e^{\lambda_{+}w}\left(\lambda_{+}\sigma -1\right)}+\delta \left(\lambda_{+}-\lambda_{-}\right)\right]. \end{array}$$

Fig. 7 gives the solution curve for the intersection of the curves such that *k*_1_ = *k*_2_. The real eigenvalue case corresponds to the blue curve in Fig. 7.

#### Matching Conditions: Complex Eigenvalues

The matching conditions at the points 0 and *w* for the complex eigenvalues case are:

$$\begin{array}{c} k_{1}=\frac{1}{2\sigma }\int_{-\infty }^{+\infty }e^{-\frac{|y-w|}{\sigma }}u\left(y,t\right)dy\\ k_{1}=\frac{\alpha }{2(\alpha +\beta )}\left[-e^{-\frac{w}{\sigma }}+1+\frac{2c\left[2c({\text{c}}_{1}f\sigma +{\text{c}}_{1})-{\text{c}}_{2}r\sigma \right]}{r\left[4{\left(cf\sigma +c\right)}^{2}+r^{2}\sigma^{2}\right]}\right]\\ +\frac{\alpha }{2(\alpha +\beta )}\left[\frac{2c{\text{c}}_{3}\;\text{exp}(-w\left(\frac{1}{\sigma }+f\right))\left[e^{fw}\left(2c(f\sigma -1)\;\text{cos}\left(\frac{rw}{2c}\right)+r\sigma \;\text{sin}\left(\frac{rw}{2c}\right)\right)-2c(f\sigma -1)e^{\frac{w}{\sigma }}\right]}{r\left[4c^{2}{\left(f\sigma -1\right)}^{2}+r^{2}\sigma^{2}\right]}\right]\\ +\frac{\alpha }{2(\alpha +\beta )}\left[\frac{2c{\text{c}}_{4}\;\text{exp}(-w\left(\frac{1}{\sigma }+f\right)\left[e^{fw}\left(2c(f\sigma -1)\;\text{sin}\left(\frac{rw}{2c}\right)-r\sigma \;\text{cos}\left(\frac{rw}{2c}\right)\right)+r\sigma e^{\frac{w}{\sigma }}\right]}{r\left[4c^{2}{\left(f\sigma -1\right)}^{2}+r^{2}\sigma^{2}\right]}\right]\\ +\frac{\alpha }{2(\alpha +\beta )}\left[\frac{2c\left[2c({\text{c}}_{1}f\sigma +{\text{c}}_{1})-{\text{c}}_{2}r\sigma \right]}{r\left[4{\left(cf\sigma +c\right)}^{2}+r^{2}\sigma^{2}\right]}\right], \end{array}$$

and

$$\begin{array}{c} k_{2}=\frac{1}{2\sigma }\int_{-\infty }^{+\infty }e^{-\frac{|y|}{\sigma }}u\left(y,t\right)dy\\ k_{2}=\frac{\alpha }{2\left(\alpha +\beta \right)}\left[\frac{\left(-e^{-\frac{w}{\sigma }}+1\right)\left[2ce^{-\frac{w}{\sigma }}\left(2c\left({\text{c}}_{1}f\sigma +{\text{c}}_{1}\right)-{\text{c}}_{2}r\sigma \right)\right]}{r\left[4{\left(cf\sigma +c\right)}^{2}+r^{2}\sigma^{2}\right]}\right]\\ +\frac{\alpha }{2(\alpha +\beta )}\left[\frac{2c{\text{c}}_{3}e^{w\left(-\left(\frac{1}{\sigma }+f\right)\right)}\left[e^{w\left(\frac{1}{\sigma }+f\right)}\left(2c(f\sigma +1)\;\text{cos}\left(\frac{rw}{2c}\right)+r\sigma \;\text{sin}\left(\frac{rw}{2c}\right)\right)-2c(f\sigma +1)\right]}{r\left[4{\left(cf\sigma +c\right)}^{2}+r^{2}\sigma^{2}\right]}\right]\\ +\frac{\alpha }{2(\alpha +\beta )}\left[\frac{2c{\text{c}}_{4}e^{w\left(-\left(\frac{1}{\sigma }+f\right)\right)}\left[e^{w\left(\frac{1}{\sigma }+f\right)}\left(2c(f\sigma +1)\;\text{sin}\left(\frac{rw}{2c}\right)-r\sigma \;\text{cos}\left(\frac{rw}{2c}\right)\right)+r\sigma \right]}{r\left[4{\left(cf\sigma +c\right)}^{2}+r^{2}\sigma^{2}\right]}\right], \end{array}$$

where

$r=\sqrt{4\beta \delta -{\left(\delta -\alpha \right)}^{2}}$, g=2β+α−δ, $f=\frac{\delta +\alpha }{2c}$, and
${\text{c}}_{1}=\text{exp}(-fw)\left[g\;\text{sin}\left(\frac{rw}{2c}\right)-r\;\text{cos}\left(\frac{rw}{2c}\right)\right]+r$
${\text{c}}_{2}=\text{exp}(-fw)\left[-g\;\text{cos}\left(\frac{rw}{2c}\right)-r\;\text{sin}\left(\frac{rw}{2c}\right)\right]+g$
${\text{c}}_{3}=g\;\text{sin}\left(\frac{rw}{2c}\right)-r\;\text{cos}\left(\frac{rw}{2c}\right)$
${\text{c}}_{4}=-g\;\text{cos}\left(\frac{rw}{2c}\right)-r\;\text{sin}\left(\frac{rw}{2c}\right)$

The part of the curve in Fig. 7 that corresponds to the complex eigenvalue case (red curve in Fig. 7) solves the equality with *k*_1_ = *k*_2_.

### Linear stability of traveling wave solutions

The linear stability of the traveling wave solutions was analyzed in detail in [97]; here, we summarize these results. To study the linear stability of the traveling wave solutions we construct a complex-valued Evans functions whose zeros determine the eigenvalues associated with the stability of the wave [98]. By obtaining the eigenvalues it is possible to determine stability (or instability) of the linearized wave. Using the Evans functions, we explore the stability of wave solutions for parameter choices restricted by the LFP data. We have shown that for some parameter settings two wave solutions exist (e.g., Fig. 6). We note that one of these wave solutions is slow and narrow, whereas the other solution is fast and wide. Moreover, the fast and wide wave has speed and width consistent with the LFP data (as illustrated in Fig. 11). Using the Evans function we find that, in the case of the fast and wide wave, the associated eigenvalues consist of eigenvalues with negative real part and the trivial zero eigenvalue (due to the translation invariance of the wave solution); this implies linear stability of the fast and wide wave. In the slow and narrow wave case, we find a positive eigenvalue (purely real) in addition to the zero eigenvalue, implying linear instability of the wave solution. For more details, please see S3 Text of Supporting Information.

### Simulations

Space was discretized using 2000 points, to represent the length of a one-dimensional path. To each of these points the differential equation system (1) was associated. Numerical simulations were written to solve these systems using a Runge-Kutta method of order four with Δ*t* = 0.005 ms. Convolutions integrals were approximated by assuming the activity was fixed within a Δ*x* interval, where Δ*x* represented a distance of 40 *μ*m. Smaller grids were also examined of Δ*x* = 20 *μ*m, and Δ*x* = 10 *μ*m, and similar results found (not shown). The waves were created by applying a 5 ms input to points in space representing 10 *μ*m. Both time and space were rescaled in order to have units of distance *x* in microns and time *t* in milliseconds.

## Supporting Information

S1 Text Activity-based model with adaptation inside of the nonlinearity. (PDF)

S2 Text Comparison of single-channel *in vivo* and model dynamics. (PDF)

S3 Text Linear stability of the traveling wave solutions. (PDF)
