# Supplementary material for: A Biologically Constrained, Mathematical Model of Cortical Wave Propagation Preceding Seizure Termination
Source: PLoS Comput Biol. 2015 Feb 17;11(2):e1004065. doi: 10.1371/journal.pcbi.1004065 (PMC4331426; doi:10.1371/journal.pcbi.1004065)
Supplement: S1 Text — (PDF) [file pcbi.1004065.s001.pdf]

Text S1: Activity-based model with adaptation inside of the nonlinearity

In the model (1) we included the adaption term outside of the Heaviside function. Here, we consider the impact of placing the adaptation term inside of the Heaviside function. To do so, we use the following formulation of an activity-based model (1b):

$$\begin{aligned} u_t(x,t) &= -\alpha u(x,t) + \alpha H\left(\frac{1}{2\sigma}\int_{-\infty}^{+\infty} e^{-\frac{|x-y|}{\sigma}} u(y,t)\,dy - \beta q(x,t) + P(x,t) - k\right) \\ q_t(x,t) &= \delta u(x,t) - \delta q(x,t) \end{aligned} \tag{1b}$$

where the parameters are as described in model (1). Note that, in this model, the adaption term $q(x,t)$ appears inside of the Heaviside function and the parameter β accounts for the strength of the adaptation. The traveling wave solutions of this model formulation are of the form:

$$u(z) = \begin{cases} c_1 e^{-\alpha z/c} & \text{if } z \geq w \\ c_2 e^{-\alpha z/c} & \text{if } 0 \leq z \leq w \\ 0 & \text{if } z \leq 0 \end{cases}$$

where w is the width of the wave, c is the speed of the wave and c_1 and c_2 are real constants that depend on the model parameters. We note in particular that this traveling wave solution goes monotonically to a rest state after excitation. This is due to the purely real eigenvalues in the associated traveling wave system. We note that this formulation does not replicate the "reverberation" observed in the clinical data, which is an important feature of the LFP waves preceding seizure termination. Replacing the Heaviside function with a smooth nonlinearity produces a similar result for this model, as we now demonstrate.

We now consider a modified model using the activity-based formulation with the adaptation term placed inside of a *smooth* nonlinearity:

$$\begin{aligned} u_t(x,t) &= -\alpha u(x,t) + \alpha S\left(\frac{1}{2\sigma}\int_{-\infty}^{+\infty} e^{-\frac{|x-y|}{\sigma}} u(y,t)\,dy - \beta q(x,t) + P(x,t) - k\right) \\ q_t(x,t) &= \delta u(x,t) - \delta q(x,t) \end{aligned} \tag{1c}$$

where the Heaviside function in (1b) has been replaced with a smooth nonlinearity - namely a sigmoid nonlinearity:

$$S(x) = \frac{1}{1 + \exp(-a_e\, x)}.$$

We note that the function $S(x)$ mimics the step function: when the variable x is small enough, the output of $S(x)$ is very near 0. The parameter a_e indicates the steepness of the sigmoid function.

We then simulated this updated model over a broad region of parameter space:

σ: (50, 100, 150, ..., 500) (in microns)
β: (0.05, 0.15, 0.25, ..., 0.55)
k: (0.05, 0.10, 0.15, ..., 0.35)
a_e: (5, 10, 15, ..., 25)

This region of parameter space was motivated by the results found in the original formulation of the model and by numerical investigation. We note that the addition of the parameter to account for sigmoid steepness (a_e) to the model formulation also implies the necessity of restricting an additional parameter from the LFP data and may not permit restriction of all model parameters in terms of just one free variable, as occurs in the original formulation of the model.

All combinations of the four parameters (σ, β, k, a_e) resulted in 2100 simulation results. We first examined each simulation to detect traveling waves. To do so, we fixed time, searched for a single broad peak of activity in space (here $x > 0.8$ for 2000 microns), and confirmed that regions outside of this peak returned to smaller values (here $0 < x < 0.8$). We also fixed space, and searched for a single broad peak of activity in time (here $x > 0.8$ for 20 milliseconds), and confirmed that times outside of this peak decreased to smaller values (here $0 < x < 0.8$). Combined, these restrictions enforce the basic properties of a traveling pulse; the activity should be large in a narrow interval of space and time, and small elsewhere. We note that these restrictions still permit detection of damped oscillations, as long as the damping is not extremely weak. With these restrictions, we detected 32 traveling waves (corresponding to 1.5% of the parameter configurations). We show the results for these 32 instances of traveling waves in Figure I of this document. In all cases, we do not find damped oscillations. These simulation results

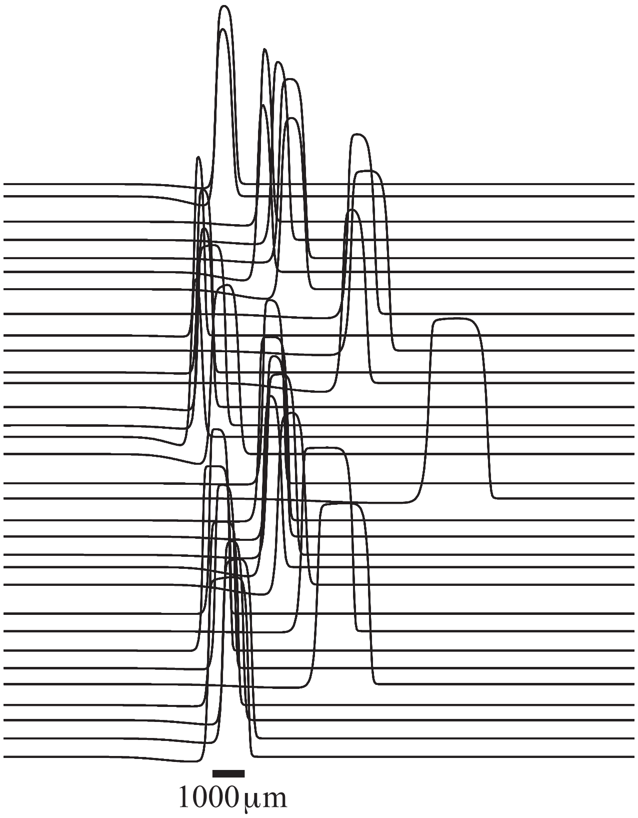

Figure I: We detected 32 traveling waves after numerical simulation of the activity-based model with the adaptation term inside of the sigmoid nonlinearity.

suggest that, if damped oscillations do exist in this model, the parameter range over which these occur is small.

We note that, with an alternative sigmoid formulation:

$$S(x) = \frac{1}{1+\exp(-a_e\, x)} - \frac{1}{1+\exp(a_e\, k)}$$

we find that traveling waves are relatively easily produced in the model (1c). Also, it is possible to find damped oscillations following the traveling waves. However, in this model, a low value of x produces a negative input to the activity dynamics. Therefore, this model differs for the original formulation,

in which low values of x result in no input. We choose here to focus on the original model formulation, in which low activity values ($x < 0$) results in no input, rather than a negative input.
