# Supplementary material for: A Biologically Constrained, Mathematical Model of Cortical Wave Propagation Preceding Seizure Termination
Source: PLoS Comput Biol. 2015 Feb 17;11(2):e1004065. doi: 10.1371/journal.pcbi.1004065 (PMC4331426; doi:10.1371/journal.pcbi.1004065)
Supplement: S2 Text — (PDF) [file pcbi.1004065.s002.pdf]

Text S2: Comparison of single-channel *in vivo* and model dynamics

To allow a more direct comparison between the LFP data and the simulation results, we examine example single channel time series from the human LFP data, and perform model simulations using the original model formulation (1), analyzing the effect of a smooth nonlinearity replacing the Heaviside function. We observe in Figure Ia that the single-channel time series for all wave events (of Seizure 1) have a similar profile, with a pulse of high activity followed by a depression of activity and a pronounced "reverberation" (i.e., small amplitude fluctuation). Using the original model (1), we obtain a prototypical wave (Figure Ib) where the features of speed and width are matched to the LFP data. We note that the solution changes abruptly from the rest state to the excited state, and it is followed by a pronounced depression of activity and a small reverberation.

We observe that the simulated wave (in Figure Ib) does not agree with all aspects of the *in vivo* data; the goal of the model is to capture some important features of the *in vivo* wave data (e.g., speed and width, and features of the reverberation). To begin to address these differences, we show in Figure Ic a simulation of the model (1) with a smooth nonlinearity (replacing the Heaviside function). We use the system:

$$\begin{aligned} u_t\left(x,t\right) = &\quad -\alpha u\left(x,t\right) + \alpha S\left(\frac{1}{2\sigma}\int_{-\infty}^{+\infty} e^{-\frac{|x-y|}{\sigma}} u\left(y,t\right)dy + P(x,t) - k\right) \quad -\alpha\beta_0 q\left(x,t\right) \\ q_t\left(x,t\right) = &\quad \delta u\left(x,t\right) - \delta q\left(x,t\right) \end{aligned} \tag{1d}$$

where the Heaviside function in (1) has been replaced with the sigmoid nonlinearity:

$$S(x) = \frac{1}{1+\exp(-a_e\, x)}.$$

Given a set of parameters (α, δ, σ, β and k) estimated from the data that produce a traveling wave solution with speed c and width w using the Heaviside nonlinearity, it is possible to find values for the parameter a_e such that the model (1d) produces a traveling wave solution. This traveling wave solution has values of speed and width that approach the estimated speed and width of the LFP wave as the parameter a_e increases (i.e., when the sigmoid is steep, and approaches the Heaviside function); see Figure I.

We note one observation from the modified model (1d): as the parameter β increases, the value of a_e necessary to obtain a traveling wave solution is higher. This result shows that, as the strength of the adaptation increases, a steeper sigmoid is required to produce traveling waves. This is due to the relationship of the parameters β and σ obtained in the matching conditions of the original model (1). In particular, as the parameter β increases the parameter σ decreases, which implies that nearby connections have a stronger effect (the connectivity function is of the form $g(x) = \frac{1}{2\sigma} \exp\left(-\frac{|x|}{\sigma}\right)$). The stronger effect of nearby connections, combined with a low value of a_e, increases the overall excitatory input in the model. In this case, the activity does not return to rest following an excitatory perturbation; instead a front of high activity arises or the system enters an oscillatory regime. Using parameter estimates determined for the case of the Heaviside nonlinearity, we are able to produce traveling waves with the sigmoid nonlinearity when the increase from rest to activity of the sigmoid function is steep enough (i.e., the parameter a_e is big enough).

We note that parameter configurations in the model (1d) near those obtained for the original model (1) (i.e., with a small increment in the excitatory threshold) produce a more accurate representation of the single channel data (see example in Figure Id). We find that, using the smooth nonlinearity in the model (1d), the wave profile is smoother, with a slower transition from rest, and also a slower transition between the active and depressed states. The profile of this wave is in better agreement with the experimental data obtained in the LFP (Figure Ia). This suggests that modifications in the adaptation (including using a smoother nonlinearity) may induce a more accurate description of the single-channel data. Additional updates to the model (e.g., the inclusion of additional intrinsic currents, or perhaps the inclusion of inhibition) may help further improve the correspondence between the model results and aspects of the *in vivo* data. However, these changes will come at the cost of an increasingly complex and analytically intractable model.

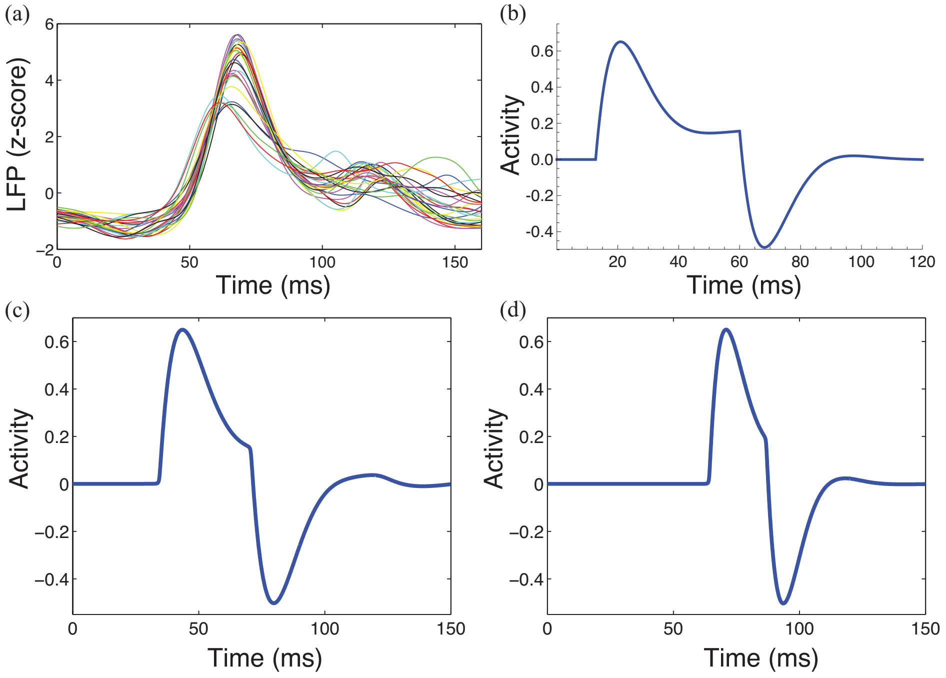

Figure I: **Example single channel times series** *in vivo* **and in simulation.** **(a)** Single-channel time series plot of the different wave events in Seizure 1 (36 wave events total). For this plot, we selected the central one-dimensional path through the microelectrode array and the electrode at the center of this one-dimensional path. **(b)** Traveling wave solution obtained from the original model formulation, using the Heaviside function. Here $\alpha = 25/\mathrm{s}$, $\delta = 2.5/\mathrm{s}$, $\beta_0 = 5$, $\sigma = 40\ \mu\mathrm{m}$ and $k = 0.07839$. **(c)** Traveling wave solution obtained using a sigmoid nonlinearity, with $\alpha = 25/\mathrm{s}$, $\delta = 2.5/\mathrm{s}$, $\beta_0 = 5$, $\sigma = 40\ \mu\mathrm{m}$, $k = 0.07839$, $a_e = 78$. The profile of the wave is similar to the Heaviside case with smoother transitions between the different states. **(d)** Another example traveling wave solution obtained using a sigmoid nonlinearity. We note that the wave solution is smoother and in more accordance with the experimental data. For this plot $\alpha = 25/\mathrm{s}$, $\delta = 2.5/\mathrm{s}$, $\beta_0 = 5$, $\sigma = 40\ \mu\mathrm{m}$, $k = 0.1176$, and a_e=72.
