# Supplementary material for: A Biologically Constrained, Mathematical Model of Cortical Wave Propagation Preceding Seizure Termination
Source: PLoS Comput Biol. 2015 Feb 17;11(2):e1004065. doi: 10.1371/journal.pcbi.1004065 (PMC4331426; doi:10.1371/journal.pcbi.1004065)
Supplement: S3 Text — (PDF) [file pcbi.1004065.s003.pdf]

Text S3: Linear stability of the traveling wave solutions.

Here we study the linearized stability of the traveling wave solutions of the model (1); a more detailed discussion of this material may be found in [2]. To do so, we study the spectrum of the linearization about the traveling wave solution. By stability, we will mean here *linear* stability. For a smooth dynamical system, having all of the spectrum of the linearized problem in the left half of the plane implies asymptotic stability. In our case, due to the discontinuous nature of the nonlinear term we cannot automatically conclude that linear stability implies nonlinear stability. We note that the linear stability results we derive below are consistent with the observed properties of the waves in the experimental data analyzed here. More specifically, we show that for certain parameter values in our model, the analysis predicts the existence of two different traveling waves, only one of which has speed and width consistent with the experimentally observed waves. Our linear stability analysis shows that:

1) The linearized equations about a wave that is not observed experimentally have a positive real eigenvalue implying that these solutions are unstable and unlikely to be observed for general initial conditions.

2) On the other hand, the linearized equations about the wave that is consistent with the experimental data has an eigenvalue at zero (corresponding to the translational invariance of the wave) but all other eigenvalues in the left half plane, showing that these waves are stable.

Linearization about the traveling wave solution

We study the spectrum of the activity-based model with adaptation previously described but with slightly different notation, that is:

$$\begin{aligned} u_t\left(x,t\right) &= -\alpha u\left(x,t\right) - \beta q\left(x,t\right) + \alpha H\left(\int_{-\infty}^{\infty} g(x-y)u\left(y,t\right)dy - k\right) \\ q_t\left(x,t\right) &= \delta u\left(x,t\right) - \delta q\left(x,t\right), \end{aligned} \tag{i}$$

where $g(x)$ is continuous, even, and $\int_{-\infty}^{\infty} g(x)dx = 1$, for example $g(x) = \frac{1}{2\sigma}e^{-\frac{|x|}{\sigma}}$ and we have set $P(x,t) = 0$ in (1). We introduce the moving frame $z = x + ct$, where $c > 0$, and look for stationary solutions of the form $v(z,t) = v(x+ct,t)$ and $p(z,t) = p(x+ct,t)$, where $v : \mathbb{R} \times \mathbb{R}_+ \to \mathbb{R}$ and

$p : \mathbb{R} \times \mathbb{R}_+ \to \mathbb{R}$. We linearize the system (i) about the stationary solution

$$V(z) = \begin{pmatrix} v(z,t) \\ p(z,t) \end{pmatrix}.$$

For this, we make the substitution $u = v(z) + \epsilon \bar{u}(z)$ and $q(z) = p(z) + \epsilon \bar{q}(z)$, where ϵ is small. We proceed to compute the derivative of each term with respect to ϵ, and then evaluate at $\epsilon = 0$. To do so, we need to consider the derivative of the Heaviside function in the sense of distributions. In particular, we obtain:

$$\left.\frac{d}{d\epsilon}\right|_{\epsilon=0} H\left(g \otimes v\left(z\right) + \epsilon g \otimes \bar{u}\left(z\right) - k\right) = \delta\left(g \otimes v\left(z\right) - k\right) g \otimes \bar{u}\left(z\right),$$

where $g \otimes u(z) = \int_{-\infty}^{\infty} g(z-y)u(y,t)dy$. In this way, we obtain the linearization of system (i), where we have made the change of variables $\bar{u} = u$:

$$\begin{pmatrix} u \\ q \end{pmatrix}_t = A \begin{pmatrix} u \\ q \end{pmatrix} - c \begin{pmatrix} u \\ q \end{pmatrix}_z + \begin{pmatrix} \alpha\delta(g \otimes v - k)(g \otimes u) \\ 0 \end{pmatrix} \quad \text{(ii)}$$

where $A = \begin{pmatrix} -\alpha & -\beta \\ \delta & -\delta \end{pmatrix}$. Similar expressions for the linearized equations of voltage-based models are studied in [4].

Recall that the interactions of neighboring cells affect the activity present at a point z, giving rise to the matching conditions previously described, that were determined by the equations $g \otimes v(0) = k$ and $g \otimes v(w) = k$. Using these conditions and properties of the delta function we transform system (ii) to:

$$\begin{pmatrix} u \\ q \end{pmatrix}_t = A \begin{pmatrix} u \\ q \end{pmatrix} - c \begin{pmatrix} u \\ q \end{pmatrix}_z + \alpha \begin{pmatrix} \frac{\delta(z) g\otimes u(0)}{h_0} + \frac{\delta(z-w) g\otimes u(w)}{h_w} \\ 0 \end{pmatrix}, \quad \text{(iii)}$$

where we have used $h_0 = |(g \otimes v)'(0)|$ and $h_w = |(g \otimes v)'(w)|$ to simplify the system. We observe that system (iii) has a forcing term involving the delta function, resulting in a discontinuous derivative at the points where the delta function is defined ($z = 0$ and $z = w$). Mathematically, in order to proceed to analyze this system, we consider the convolution of both sides of the equation with a continuous function. For our purposes, and motivated by the coefficients of the inhomogeneous term in (ii) we proceed to convolve system (iii) with the kernel $g(z)$. Again using properties of the delta function we obtain:

$$\left(\begin{array}{c} g \otimes u \\ g \otimes q \end{array} \right)_t = A \left(\begin{array}{c} g \otimes u \\ g \otimes q \end{array} \right) - c \left(\begin{array}{c} g \otimes u \\ g \otimes q \end{array} \right)_z + \alpha \left(\begin{array}{c} \frac{g(z) g\otimes u(0)}{h_0} + \frac{g(z-w) g\otimes u(w)}{h_w} \\ 0 \end{array} \right). \tag{iv}$$

We now have a non-autonomous system of equations for the quantities $g \otimes u$ and $g \otimes q$ which has *continuous* coefficients.

Localization of the spectrum

We now proceed to determine the spectrum of system (iv). To do so, we consider the eigenvalue problem $(g \otimes U)_t = \lambda(g \otimes U)$, where $\lambda \in \mathbb{C}$. This gives rise to the system:

$$\left(\begin{array}{c} g \otimes u \\ g \otimes q \end{array} \right)_z = A(\lambda) \left(\begin{array}{c} g \otimes u \\ g \otimes q \end{array} \right) + \frac{\alpha}{c} \left(\begin{array}{c} \frac{g(z)}{h_0} g \otimes u(0) + \frac{g(z-w)}{h_w} g \otimes u(w) \\ 0 \end{array} \right) \tag{v}$$

where $A(\lambda) = \frac{1}{c} A - \lambda I$ and we have scaled λ by c.

Since the last term in (v) has coefficients that go to zero as $z \to \pm\infty$ and since it contains no derivatives, the essential spectrum will be determined by the constant coefficient part of the operator. In this way, we can determine the essential spectrum by Fourier transforming the operator, obtaining that it consists of two vertical lines with $Re(\lambda) = -(\frac{\alpha+\delta}{2c}) \pm \frac{1}{2c}\sqrt{(\alpha-\delta)^2 - 4\delta\beta}$, where α, δ, c and β are all positive. Thus, the essential spectrum of the linearized equations lies strictly in the left half plane and the instabilities can arise only through the eigenvalues which we now locate with the aid of the Evans function.

Construction of an Evans function

We now study the point spectrum of the wave. To do so, we construct an Evans function for λ in the region Ω, where

$$\Omega = \{\lambda \in \mathbb{C} \mid \text{eigenvalues of } A(\lambda) \text{ have strictly negative real part}\}.$$

Due to the previous result we have that Ω contains the positive half plane. The roots of the Evans function will be in correspondence with the point eigenvalues of the linearization about the wave [1]. Following the methodology of [3], we consider the system (v) and we use the variation of parameters

formula to obtain the solution of system (v):

$$g \otimes U(z) = e^{A(\lambda)z} \left[g \otimes U(z_0) + \int_{z_0}^{z} \frac{\alpha}{c} e^{-A(\lambda)y} \begin{pmatrix} \frac{g(y)}{h_0} g \otimes u(0) + \frac{g(y-w)}{h_w} g \otimes u(w) \\ 0 \end{pmatrix} dy \right]. \tag{vi}$$

By assumption, the eigenvalues of $A(\lambda)$ have negative real part in the region Ω. Thus when looking for a bounded solution of $g \otimes U$ we obtain:

$$\begin{pmatrix} g \otimes u \\ g \otimes q \end{pmatrix} = \begin{pmatrix} \int_{-\infty}^{z} \frac{\alpha}{c} e_{1,1}^{A(\lambda)(z-y)} \left(\frac{g(y)}{h_0} g \otimes u(0) + \frac{g(y-w)}{h_w} g \otimes u(w) \right) dy \\ \int_{-\infty}^{z} \frac{\alpha}{c} e_{2,1}^{A(\lambda)(z-y)} \left(\frac{g(y)}{h_0} g \otimes u(0) + \frac{g(y-w)}{h_w} g \otimes u(w) \right) dy \end{pmatrix} \tag{vii}$$

where $e_{i,j}^{A(\lambda)(z-y)}$ is the entry in the ith-row and jth-column of the matrix exponential $e^{A(\lambda)(z-y)}$. Substituting $z = 0$ into both sides of (vii) gives a pair of linear equations linking $g \otimes u(0)$, $g \otimes u(w)$, $g \otimes q(0)$, $g \otimes q(w)$. Likewise, substituting $z = w$ into both sides of (vii) gives two more linear equations linking these quantities. If this system of linear equations has a (non-zero) solution, then our eigenfunction equation (vi) has a non-zero solution for that value of λ, i.e., λ is an eigenvalue. This system of equations in turn has a non-zero solutions if and only if the determinant of its coefficient matrix vanishes. We define this determinant to be the Evans function. In this case, it can be computed explicitly and we find:

$$\begin{aligned} E(\lambda) = &\left(1 - \frac{\alpha}{ch_0} \int_{-\infty}^{0} e_{1,1}^{A(\lambda)(-y)} g(y) dy \right) \left(1 - \frac{\alpha}{ch_w} \int_{-\infty}^{w} e_{1,1}^{A(\lambda)(w-y)} g(y-w) dy \right) \\ &- \frac{\alpha^2}{c^2 h_0 h_w} \left(\int_{-\infty}^{w} e_{1,1}^{A(\lambda)(w-y)} g(y) dy \right) \left(\int_{-\infty}^{0} e_{1,1}^{A(\lambda)(-y)} g(y-w) dy \right) \end{aligned} \tag{viii}$$

Stability of a wave with features consistent with the observed LFP waves.

We proceed to numerically analyze the constructed Evans function for parameter values consistent with the results obtained in the main manuscript. In particular, we focus on the complex eigenvalue case, that is when $\beta > \frac{(\alpha-\delta)^2}{4\delta}$. For the following example we fix α=25 Hz, δ = 2.5 Hz, σ=160 μm and β_0 = 2.1 (β=$\beta_0\alpha$) and explore the stability of waves as we vary the parameter k. In Figure I we obtain the existence of two waves if we fix k = 0.19. In particular, the "fast" wave has speed c_{fast}=220 μm/ms and width w_{fast}=1896 μm; and the "slow" wave has speed c_{slow}=59 μm/ms

and width w_{slow}=138 μm. We note that the fast and wide wave has speed and width consistent with the observed LFP waves. Thus, we intuitively expect this wave to be stable. Since we have explicit formulas for the matrix components that appear in (viii) we can substitute in the values of w and c corresponding to each of these waves and evaluate the Evans function numerically. The results of this evaluation are shown in Figures Ic,d where we display contour plots of the real and imaginary parts of $E(\lambda)$ for λ in the positive half plane. For the slow wave we see that there is a positive, real zero of the Evans function. This implies that the linearized equations have a positive eigenvalue and hence this wave is unstable. In Figure Ib we analyze the change of this eigenvalue as the parameter k increases. For the fast wave (Figure Id), the only zero of the Evans function occurs at $\lambda = 0$. This corresponds to the translation invariance of the wave. Since all other eigenvalues occur in the left half plane, this corresponds to a linearly stable solution.

References

[1] J.C. Alexander, R.A. Gardner, and C.K.R.T. Jones. A topological invariant arising in the stability analysis of travelling waves. *Journal fur die reine und angewandte Mathematik*, 410:167–212, 1990.

[2] L.R. Gonzalez-Ramirez. *Existence and stability of traveling waves in a biologically constrained model of seizure wave propagation.* PhD thesis, Boston University, 2014.

[3] B. Sandstede. Evans functions and nonlinear stability of traveling waves in neuronal network models. *International Journal of Bifurcation and Chaos*, 17:2693–2704, 2007.

[4] Linghai Zhang. Existence, uniqueness and exponential stability of traveling wave solutions of some integral differential equations arising from neuronal networks. *Journal of Differential Equations*, 197:162–196, 2004.

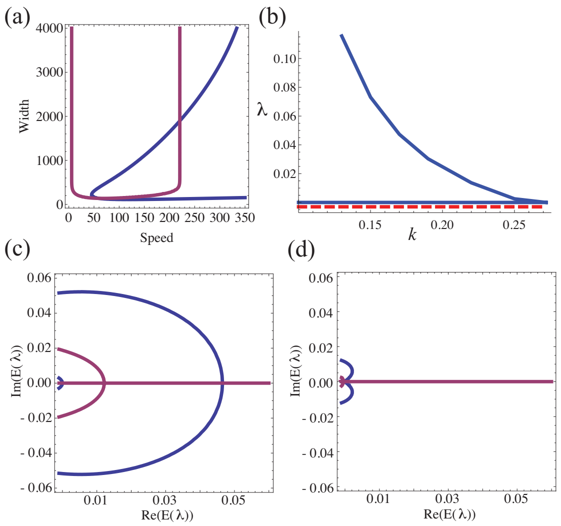

Figure I: **Examination of stability in the neural model. (a)** The blue and purple curves indicate the matching conditions at the points 0 and w, respectively. We fix $\alpha =$ 25 Hz, δ=2.5 Hz, σ=160 μm, β_0=2.1 and $k = 0.19$. The two matching condition curves intersect at two points, resulting in a "fast" wave and a "slow" wave. **(b)** Change of the (purely) real eigenvalue λ of the slow wave, as the synaptic threshold k increases. We note that, as k increases, the real eigenvalue decreases until a bifurcation occurs at $k = 0.265$; beyond this value of k the model no longer supports traveling wave solutions for the chosen parameters. **(c)** Zero contours of the real (blue line) and imaginary (purple line) parts of the Evans function for the slow wave. The intersection of both curves suggest a zero eigenvalue and a positive real part eigenvalue. **(d)** Zero contours of the real (blue line) and imaginary (purple line) parts of the Evans function for the fast wave. The intersection of both lines at zero shows the existence of a zero eigenvalue at zero. No other positive real part eigenvalues exist in the neighborhood of zero.
